# Histone lysine methyltransferase SMYD3 promotes oral squamous cell carcinoma tumorigenesis via H3K4me3-mediated HMGA2 transcription

**DOI:** 10.1186/s13148-023-01506-9

**Published:** 2023-05-26

**Authors:** Zongcheng Yang, Fen Liu, Zongkai Li, Nianping Liu, Xinfeng Yao, Yu Zhou, Liyu Zhang, Pan Jiang, Honghong Liu, Lingming Kong, Chuandong Lang, Xin Xu, Jihui Jia, Takahito Nakajima, Wenchao Gu, Lixin Zheng, Zhihong Zhang

**Affiliations:** 1 Department of Stomatology, The First Affiliated Hospital of USTC, Division of Life Sciences and Medicine, University of Science and Technology of China, Hefei, Anhui People’s Republic of China; 2 Department of Clinical Laboratory, Linyi Central Hospital, Linyi, Shandong People’s Republic of China; 3 Department of General Surgery, The First Affiliated Hospital of USTC, Division of Life Sciences and Medicine, University of Science and Technology of China, Hefei, Anhui People’s Republic of China; 4 Department of Orthopedics, The First Affiliated Hospital of USTC, Division of Life Sciences and Medicine, University of Science and Technology of China, Hefei, Anhui People’s Republic of China; 5 Department of Implantology, School and Hospital of Stomatology, Cheeloo College of Medicine, Shandong University, Shandong Key Laboratory of Oral Tissue Regeneration, Shandong Engineering Laboratory for Dental Materials and Oral Tissue Regeneration, Jinan, Shandong People’s Republic of China; 6 Department of Microbiology/Key Laboratory for Experimental Teratology of the Chinese Ministry of Education, School of Basic Medical Science, Cheeloo College of Medicine, Shandong University, Jinan, Shandong People’s Republic of China; 7 Department of Diagnostic and Interventional Radiology, University of Tsukuba, Tsukuba, Ibaraki Japan; 8 Department of Diagnostic Radiology and Nuclear Medicine, Gunma University Graduate School of Medicine, Maebashi, Gunma Japan

**Keywords:** Oral squamous cell carcinoma, Tumorigenesis, Epigenetics, SMYD3, HMGA2

## Abstract

**Background:**

Epigenetic dysregulation is essential to the tumorigenesis of oral squamous cell carcinoma (OSCC). SET and MYND domain-containing protein 3 (SMYD3), a histone lysine methyltransferase, is implicated in gene transcription regulation and tumor development. However, the roles of SMYD3 in OSCC initiation are not fully understood. The present study investigated the biological functions and mechanisms involved in the SMYD3-mediated tumorigenesis of OSCC utilizing bioinformatic approaches and validation assays with the aim of informing the development of targeted therapies for OSCC.

**Results:**

429 chromatin regulators were screened by a machine learning approach and aberrant expression of SMYD3 was found to be closely associated with OSCC formation and poor prognosis. Data profiling of single-cell and tissue demonstrated that upregulated SMYD3 significantly correlated with aggressive clinicopathological features of OSCC. Alterations in copy number and DNA methylation patterns may contribute to SMYD3 overexpression. Functional experimental results suggested that SMYD3 enhanced cancer cell stemness and proliferation in vitro and tumor growth in vivo. SMYD3 was observed to bind to the High Mobility Group AT-Hook 2 (HMGA2) promoter and elevated tri-methylation of histone H3 lysine 4 at the corresponding site was responsible for transactivating HMGA2. SMYD3 also was positively linked to HMGA2 expression in OSCC samples. Furthermore, treatment with the SMYD3 chemical inhibitor BCI-121 exerted anti-tumor effects.

**Conclusions:**

Histone methyltransferase activity and transcription-potentiating function of SMYD3 were found to be essential for tumorigenesis and the SMYD3–HMGA2 is a potential therapeutic target in OSCC.

**Supplementary Information:**

The online version contains supplementary material available at 10.1186/s13148-023-01506-9.

## Background

Incidence of oral squamous cell carcinoma (OSCC), a major subtype of head and neck squamous cell carcinoma (HNSCC), has increased over the past few decades but many OSCC patients are diagnosed at advanced stages [1–3]. There have been significant advances in targeted therapies and immunotherapies but OSCC patients continue to suffer from psychological distress and compromised quality of life, contributing to high suicide and reduced survival rates [3–5]. Thus, there is an acute need to explore molecular mechanisms of OSCC tumorigenesis and identify novel therapeutic targets.

Tumorigenesis is a multistep process resulting from genetic mutations and epigenetic modifications, collectively referred to as oncogenic transformation [6, 7]. Epigenetic dysregulation, which involves alterations in histone modifications, DNA methylation, and non-coding RNAs, has an impact on the self-renewal capabilities and unlimited proliferation of cancer cells [8, 9]. Chromatin regulators are a group of enzymes with specific functional domains that recognize and change the epigenetic state, influencing gene replication and transcription in a cellular environment-dependent manner [10]. Genomic changes or aberrant expression of chromatin regulators are widely observed in tumors, allowing remodeling of transcriptional networks and cellular reprogramming [11]. Chromatin regulators influence cell cycle progression, maintenance of cell stemness, constitutive activation of cell signaling pathways and tumor microenvironment composition in OSCC [12, 13].

SET and MYND domain-containing protein 3 (SMYD3), a histone lysine methyltransferase, is a chromatin regulator with oncogenic activity [14]. The methylating activity of SMYD3 on histone H3 lysine 4 (K4) in the presence of HSP90A was detected in 2004. SMYD3 complexed with RNA polymerase II and bound 5′-CCCTCC-3′ and 5′-GGAGGG-3′ motifs in the promoter region to transcriptionally activate downstream genes, like Nkx2.8 in colorectal and hepatocellular carcinomas [15]. SMYD3 has been implicated as a transcriptional activator in lung, kidney, ovarian, prostate and breast cancers [14, 16]. The long non-coding RNA, LTSCCAT, facilitated tongue squamous cell carcinoma invasion and metastasis via targeting the miR-103a-2-5p/SMYD3/Twist1 axis [17]. Nascent tumorigenic cells have been found to activate the SMYD3–H3K4m3 pathway to upregulate CSDE1 expression and stabilize phosphatase, TCPTP, promoting STAT1 dephosphorylation and weakening the immunogenic phenotype of tumor-repopulating cells [18]. In diffuse large B-cell lymphoma (DLBCL), SMYD3 bound specific sequences of PKM2 and promoted DLBCL cell proliferation and aerobic glycolysis via H3K4me3-mediated PKM2 transcription [19]. SMYD3 inhibitors have been found to be effective in certain anti-tumoral therapies [20, 21]. However, the molecular mechanisms through which SMYD3 contributes to tumorigenesis in OSCC remain obscure.

The potential causative relationship between chromatin regulators and OSCC was explored using a data-driven paradigm and the involvement of SMYD3 as a potential driver gene in OSCC tumorigenesis was investigated. SMYD3 exhibited copy number variation (CNV), aberrant DNA methylation and abnormally elevated expression in OSCC. SMYD3 overexpression was associated with unfavorable prognosis in OSCC patients. High Mobility Group AT-Hook 2 (HMGA2) has been found to be involved in the maintenance of cancer stem cell properties and regulates HNSCC/OSCC development and progression [22, 23]. SMYD3 enhanced HMGA2 transcription by binding to a specific site on the promoter and increasing H3K4me3 modification to promote stemness maintenance and malignant proliferation and facilitate OSCC initiation during the present work. Targeting of SMYD3 by inhibitor, BCI-121, has potential for clinical treatment. New epigenetic mechanisms and a theoretical basis for intervention using anti-tumor drugs targeting SMYD3 in OSCC are presented.

## Results

### Deregulation and prognostic value of chromatin regulators in OSCC

**Fig. 1:**
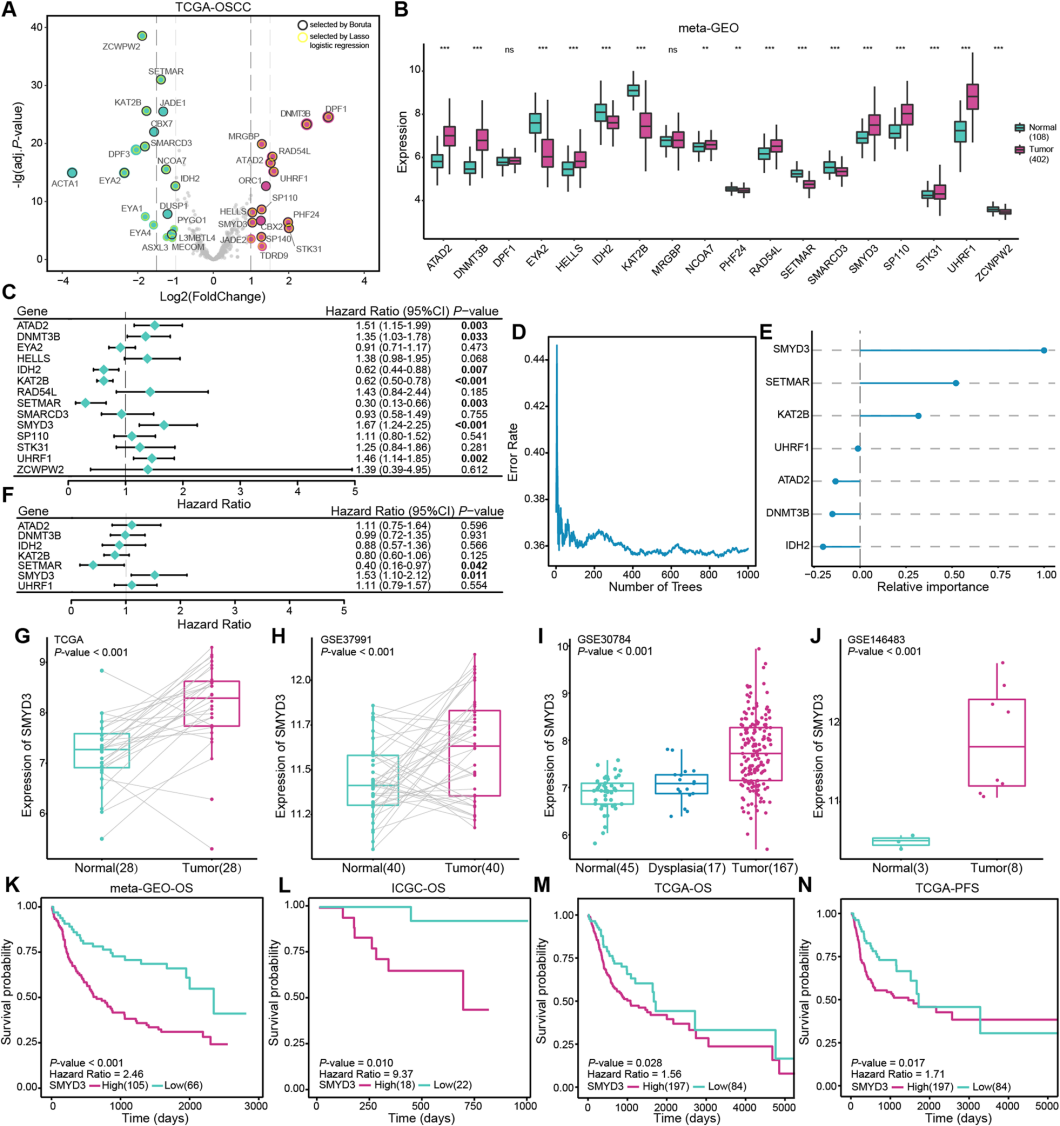
Deregulation of chromatin regulators in OSCC. **A** Volcano plots showing the 34 DEGs between normal (n = 29) and OSCC (n = 281) samples in the TCGA database and clarifying the 19 chromatin regulators were closely related to OSCC tumorigenesis by Lasso logistic regression and Boruta machine learning algorithm. **B** Differential expression profiles of 14 chromatin regulators in normal (n = 108) and OSCC (n = 402) samples from the meta-GEO dataset (SP140 was not present on the microarray chip). **C** Univariate Cox regression analyses of the 14 chromatin regulators in the meta-GEO dataset. Hazard Ratio and *P*-values were displayed. **D**,** E** Random survival forest analysis, where the error rate was reduced to a stable value as the number of trees increases, and genes were ranked according to importance. **F** Multivariate Cox regression analyses of the 7 chromatin regulators in the meta-GEO dataset. Hazard Ratio and *P*-values were displayed. **G–J** Boxplot indicating SMYD3 expression in different tissue samples and cell lines from the TCGA database, GSE37991 dataset, GSE30784 dataset and GSE146483 dataset. Numbers in parentheses indicate the sample size. **K–N** Up-regulation of SMYD3 had a significantly shorter OS time and PFS time in the meta-GEO dataset, ICGC database and TCGA database. Numbers in parentheses indicate the sample size. *P*-values were obtained from the log-rank tests. Ns, not significant, *P < 0.05, **P ≤ 0.01, and ***P ≤ 0.001

To investigate the roles of the chromatin regulators in OSCC, we assessed the overlap of the chromatin regulators in the CR2Cancer (http://cis.hku.hk/CR2Cancer/) and TCGA databases, and 401 epigenetic genes were identified. To identify the genes closely related to OSCC, we specifically examined the expression of the 401 chromatin regulators and performed differential expression analysis in the TCGA database. Thirty-four DEGs between normal and tumor samples were gained and introduced into the feature selection processes (Fig. 1A). Specifically, we leveraged Lasso logistic regression and Boruta random forest algorithms to perform dimension reduction to extract the 19 chromatin regulators from the 34 DEGs (Fig. 1A). Most of these 19 genes were aberrantly expressed in tumor samples compared with normal samples in the meta-GEO dataset. Fourteen genes with consistent validation results were retained (SP140 was not present on the microarray chip; Fig. 1B). We investigated the prognostic value of these 14 genes in the meta-GEO dataset. Univariate Cox regression analysis revealed that altered expression of ATAD2, DNMT3B, IDH2, KAT2B, SETMAR, SMYD3, and UHRF1 could impact patient prognosis (Fig. 1C). Random survival forest plots (the relationship between the error rate and the number of classification trees was shown in Fig. 1D) revealed that SMYD3 ranked first in its effect on patient survival (Fig. 1E). The multivariate Cox regression analysis also indicated that SETMAR and SMYD3 could be regarded as independent prognostic factors (Fig. 1F). Thus, we speculated that the occurrence of OSCC might be correlated with the deregulation of chromatin regulators, especially the upregulation of SMYD3.

### SMYD3 is upregulated in OSCC, and overexpressed SMYD3 indicates a poor prognosis

Previous studies suggested that SMYD3 participates in the development and progression of various tumors by regulating activation and repression of a series of genes and proteins [14]. We firstly assessed SMYD3 expression in OSCC. Our analysis of OSCC samples paired with adjacent normal tissues from the TCGA database and GSE37991 dataset indicated that SMYD3 expression was elevated significantly in OSCC samples (Fig. 1G, H). Furthermore, the expression levels of SMYD3 increased sequentially during OSCC tumorigenesis (Fig. 1I). In addition, SMYD3 expression was significantly higher in OSCC cell lines than in normal oral epithelial cell lines (Fig. 1J). To verify the diagnostic significance of SMYD3 for OSCC, ROC curve analysis was performed in the TCGA (all OSCC samples), meta-GEO, TCGA (paired samples), GSE37991, and GSE30784 datasets, showing good average area under curve (AUC) of 0.801, 0.771, 0.839, 0.833 and 0.850 respectively (Additional file 1: Fig. S1A–E). Kaplan–Meier analysis results from different datasets revealed that elevated SMYD3 expression was associated with poor OS and PFS for OSCC (Fig. 1K–N).

**Fig. 2:**
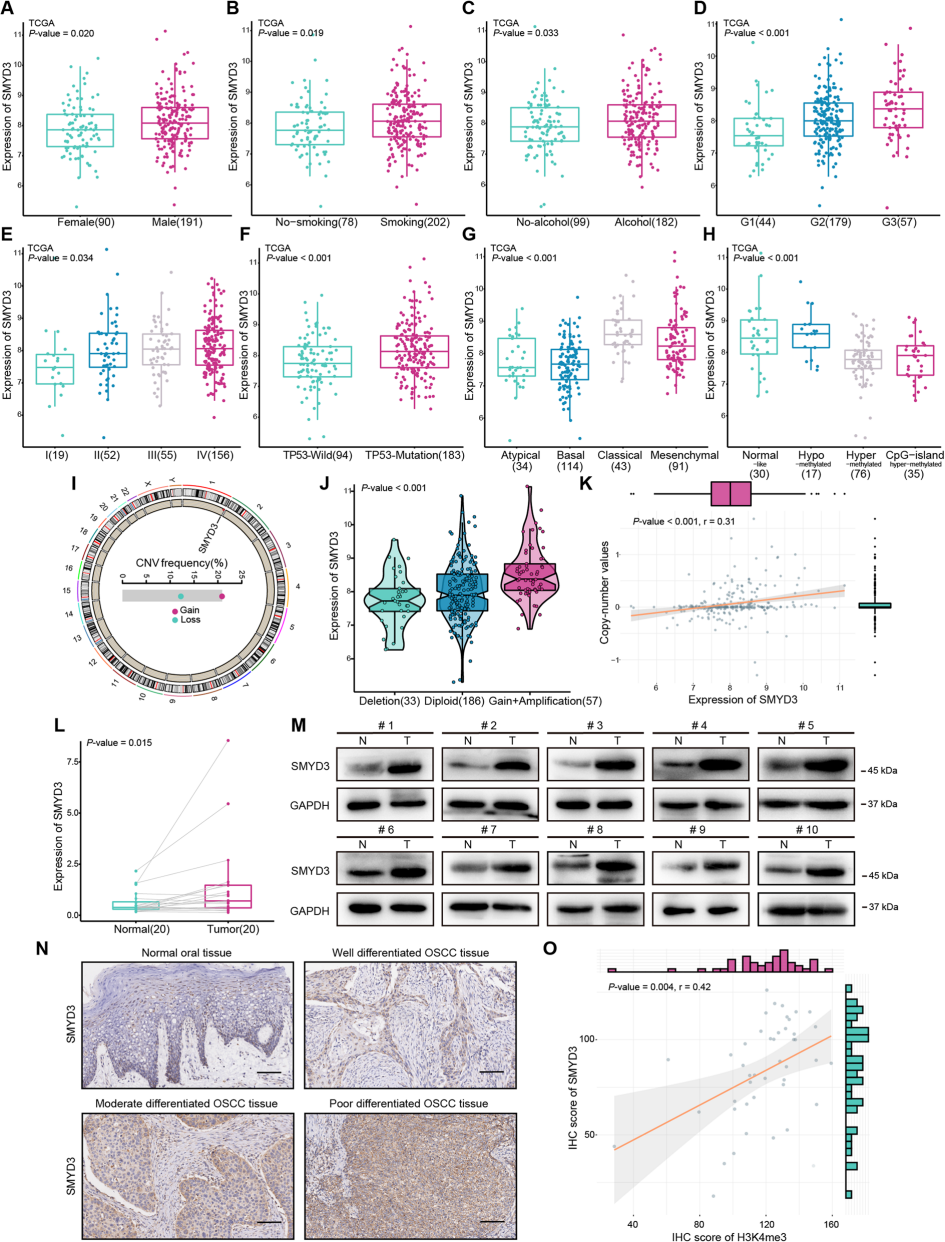
SMYD3 is upregulated in OSCC samples. **A–H** The box plots showed the distribution of SMYD3 expression in subtypes of TCGA-OSCC cohort. Numbers in parentheses indicate the sample size. **I** The location of CNV of SMYD3 on 23 chromosomes using the TCGA-OSCC cohort. **J** Deletion, diploid, copy number gain and amplification were involved in the deregulation of SMYD3 expression as analyzed by cBioPortal using TCGA-OSCC data. Numbers in parentheses indicate the sample size. **K** Correlation between SMYD3 CNV and mRNA expression. **L** Quantitative result of qRT-PCR of SMYD3 in 20 paired adjacent normal and OSCC tissues. **M** The protein levels of SMYD3 in 10 pairs of OSCC tissues (T) and adjacent normal tissues (N) measured by Western blotting. **N** Images of IHC staining for SMYD3 in normal tissues and different histologic grades of OSCC tissues (n = 131, from Stomatological Hospital of Shandong University and Shanghai Qutdo Biotech Company). Scale bars: 50 μm. **O** Quantification of SMYD3 IHC staining was correlated with that of H3K4me3 IHC staining in OSCC samples (n = 45, from Shanghai Qutdo Biotech Company)

The clinical significance of SMYD3 was further assessed in OSCC samples from the TCGA database. Elevated SMYD3 expression was significantly correlated with male, patients with a history of smoking and alcohol consumption, advanced histologic grade and TNM stage, the TP53-mutation group, and classical and hypo-methylated subtypes (Fig. 2A–H). Genomic analyses revealed that SMYD3 exhibited a widespread frequency of CNV. The location of CNVs of SMYD3 on chromosomes is shown in Fig. 2I. Notably, the copy number gain and amplification of SMYD3 were frequently observed in OSCC tissues (Fig. 2I, J), and the copy number was significantly correlated with mRNA expression level (*P* < 0.001, r = 0.31; Fig. 2K). ﻿ Additionally, we observed a negative correlation between SMYD3 mRNA expression and methylation levels on particular probes, such as cg06985779 and cg15962031 (Additional file 1: Fig. S2A), with a concomitant reduction in DNA methylation levels of SMYD3 in tumor samples (Additional file 1: Fig. S2B). We also noted a relatively low frequency of SMYD3 somatic mutations (Additional file 1: Fig. S2C). These results suggested that SMYD3 upregulation in OSCC could be attributed in part to the CNV status and DNA methylation alterations.

To validate these findings, we evaluated the data from qRT-PCR, Western blotting, and IHC staining in our clinical samples. The mRNA and protein levels of SMYD3 were higher in the OSCC samples than in paired adjacent normal tissues (Fig. 2L, M). IHC staining revealed that SMYD3 was overexpressed in OSCC tissues and the protein expression increased as the malignant tumors progressed (Fig. 2N). The AUC values obtained by ROC curve analyses from qRT-PCR and IHC staining were 0.879 and 0.765, which held the significance to support the diagnostic value of SMYD3 for OSCC (Additional file 1: Fig. S2D, E). SMYD family normally catalyses H3K4me3, which predominantly associates with active promoters [6]. Notably, a positive correlation was observed in OSCC samples between H3K4me3 level and SMYD3 expression (n = 45,* P* = 0.004, r = 0.42; Fig. 2O, Additional file 1: Fig. S3A–D). These results reinforced the idea that SMYD3 might be an oncogene and a potential biomarker in OSCC.

### SMYD3 correlates with malignant transformation of epithelial cells in OSCC

**Fig. 3:**
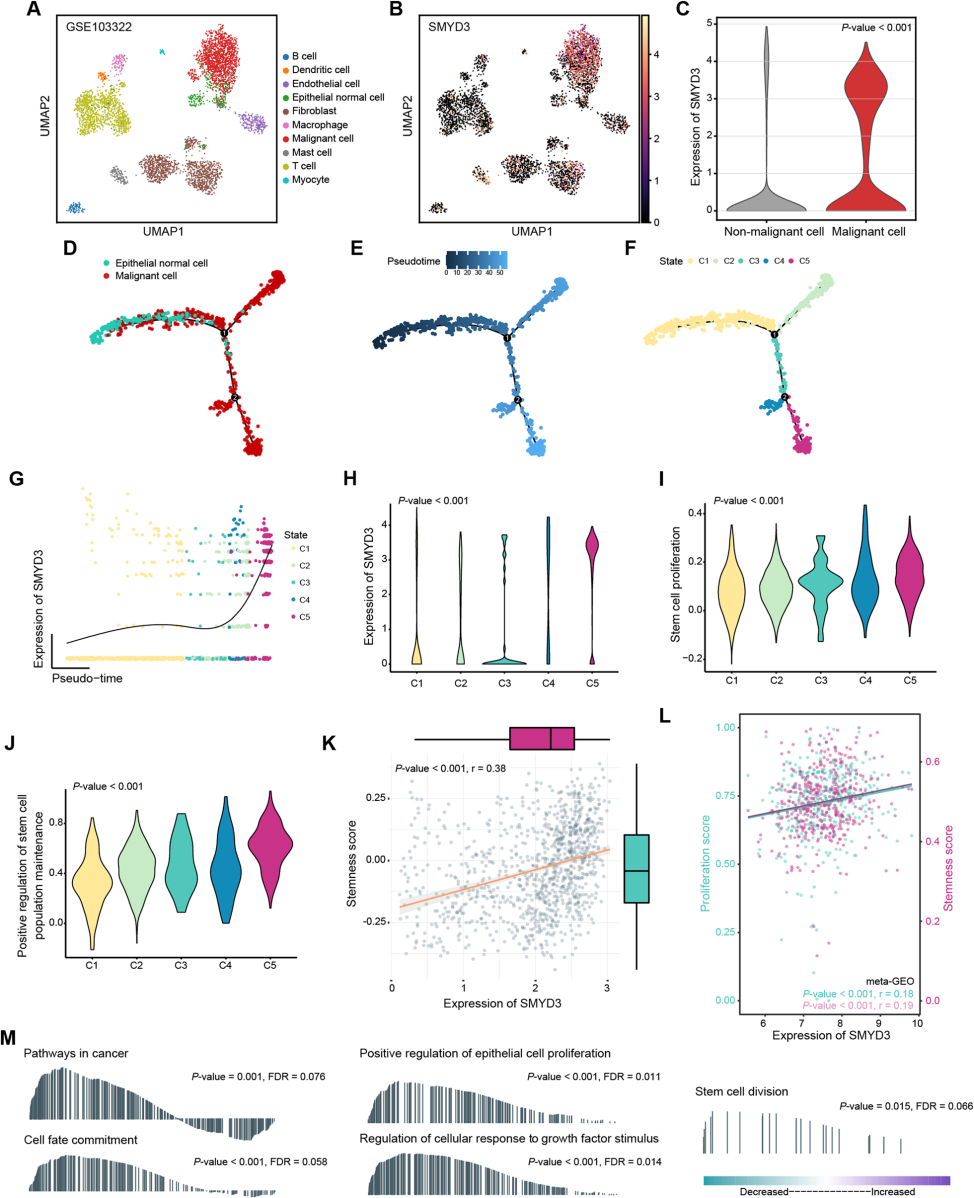
SMYD3 is overexpressed in malignant epithelial cells and correlates with cell stemness in OSCC. **A** UMAP dimensionality reduction was used to show the distribution and dissimilarity of cell types in GSE103322. **B**, **C** SMYD3 is highly expressed in malignant cells. **D**, **E** A pseudotime trajectory was plotted to describe the evolution of epithelial cells, and the progression trajectory originated from normal epithelial cells and developed into two main branches. **F** Epithelial cells including normal and malignant cells were segmented into five clusters defined as C1–C5. **G**, **H** In the (dynamic) expression profile of SMYD3 in epithelial cells pseudotime, SMYD3 became highly expressed in C5 group. **I**, **J** Violin plots showed the level of “stem cell proliferation” and “positive regulation of stem cell population maintenance” in C1–C5 of epithelial cells. **K** In malignant cells from single-cell RNA-seq dataset (GSE103322), SMYD3 expression was positively correlated with cell stemness score measured by GSVA. **L** SMYD3 expression was positively correlated with cell stemness as well as proliferation score measured by ssGSEA in meta-GEO dataset. **M** The GSEA results of RNA-seq on CAL-27 transfected with NC and SMYD3 siRNA groups

To clarify the expression profile and potential biological functions of SMYD3 in OSCC development, single-cell RNA-seq data were analyzed. The dataset (GSE103322) covers both malignant and non-malignant cells isolated from oral cavity HNSCC tumors [24]. We analysed the data from primary tumors and UMAP reduction was utilized to illustrate the distribution of cell types according to the metadata (Fig. 3A), and SMYD3 was highly expressed in malignant cells (Fig. 3B, C). Next, we performed pseudotime trajectory analysis to describe the evolution of epithelial cells, and the pseudotime value indicated the putative developmental directions (Fig. 3D, E). As shown in Fig. 3F, epithelial cells including normal and malignant cells were segmented into five clusters defined as C1–C5, and the progression trajectory originated from normal epithelial cells and developed into two main branches being ended with C5 and C2. In the dynamic expression profile, SMYD3 was found to be highly expressed at the final stage (C5; Fig. 3G, H). Interestingly, violin plots showed that the scoring of “stem cell proliferation” and “positive regulation of stem cell population maintenance” in C5 were highest among all clusters (Fig. 3I, J). To elucidate this suggestive finding, we performed correlation analysis using single-cell RNA-seq malignant cell data and found that SMYD3 expression was positively correlated with the cell stemness score (*P* < 0.001, r = 0.38; Fig. 3K). Similar results were obtained for SMYD3 expression, cell stemness, and the proliferation score in the meta-GEO dataset (Fig. 3L). Next, RNA-seq on CAL-27 transfected with NC and SMYD3 siRNA was leveraged and identified 348 DEGs between the two groups (Additional file 2: Table S1). GO and KEGG analyses revealed that these DEGs (excluding SMYD3) were associated with the regulation of gene transcription, cell differentiation, cell proliferation regulation, stem cell differentiation, cell growth factors, and tumor-associated signalling pathways (Additional file 1: Fig. S4A, B). In addition, the GSEA results suggested that cancer pathways, positive regulation of epithelial cell proliferation, cell fate commitment, regulation of cellular responses to growth factor stimulus, and stem cell division were significantly enriched in the NC group compared with the SMYD3 knockdown group (Fig. 3M). These results indicated that SMYD3 might regulate the maintenance of OSCC cell stemness and cell proliferation, thereby promoting tumorigenesis.

### SMYD3 facilitates OSCC cell stemness maintenance and proliferation in vitro and tumorigenesis in vivo

**Fig. 4:**
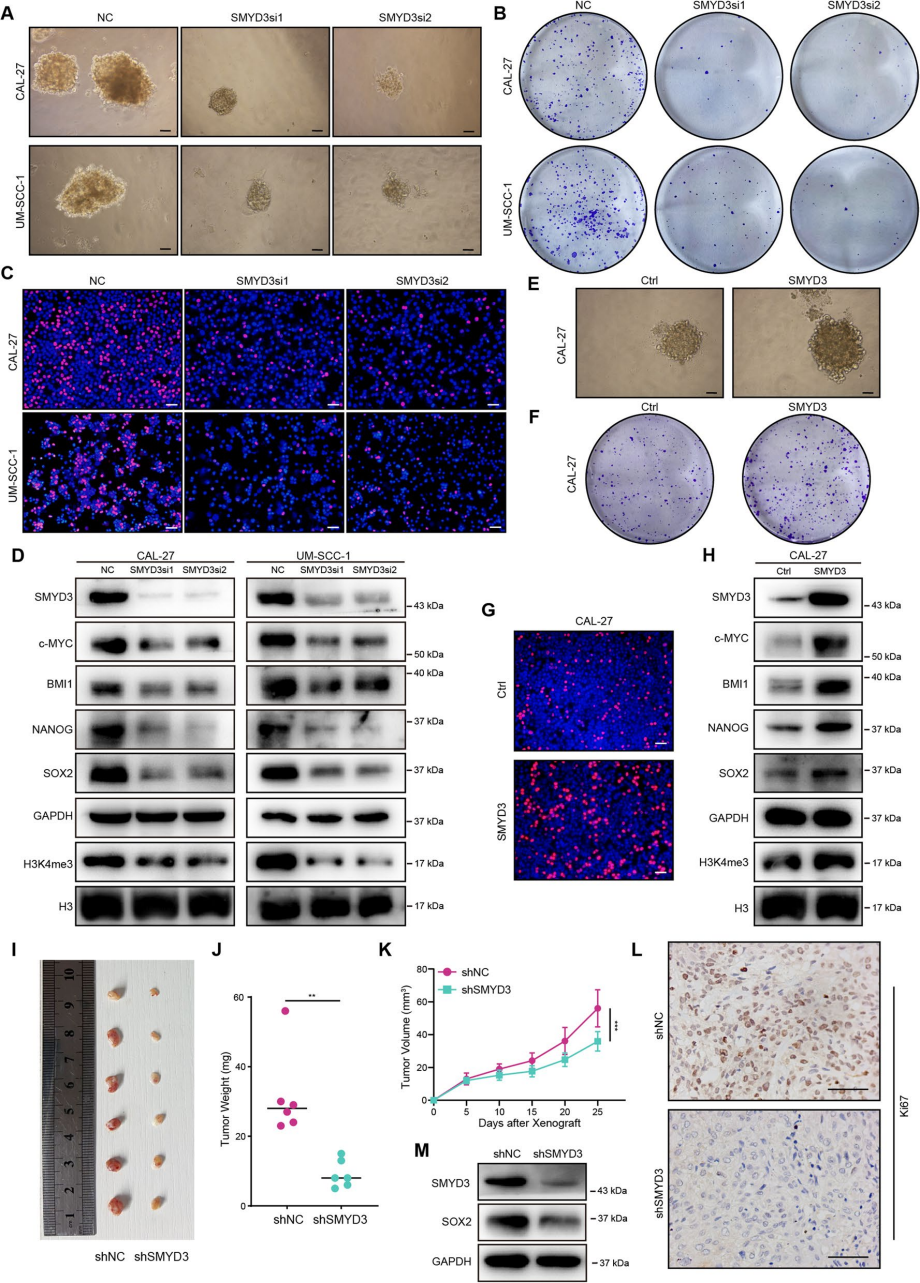
SMYD3 facilitates OSCC cell stemness maintenance and proliferation in vitro and tumorigenesis in vivo. **A** Representative image of NC- or SMYD3 siRNA-transfected CAL-27 and UM-SCC-1 cells in a secondary tumorsphere formation assay. Scale bars: 50 μm. **B** Representative images of NC- or SMYD3 siRNA-transfected CAL-27 and UM-SCC-1 cells in a colony formation assay. **C** Representative images of NC- or SMYD3 siRNA-transfected CAL-27 and UM-SCC-1 cells in an EdU staining assay. Scale bars: 50 μm. **D** Western blotting analyses showing that SMYD3, c-MYC, BMI1, NANOG, SOX2 and H3K4me3 protein expression were decreased in CAL-27 and UM-SCC-1 cells. **E** Representative images of vector- or SMYD3 plasmid-transfected CAL-27 cells in a secondary tumorsphere formation assay. Scale bars: 50 μm. **F** Representative images of vector- or SMYD3 plasmid-transfected CAL-27 cells in a colony formation assay. **G** Representative images of vector- or SMYD3 plasmid-transfected CAL-27 cells in an EdU staining assay. Scale bars: 50 μm. **H** Western blotting analyses showing that SMYD3, c-MYC, BMI1, NANOG, SOX2 and H3K4me3 protein expression were elevated in SMYD3 plasmid-transfected CAL-27 cells. **I** Subcutaneous tumor formation in nude mice of shNC and shSMYD3 groups (n = 6/group). **J**, **K** Tumor weight and tumor growth curves in the nude mouse xenograft model. **L** IHC staining for Ki67 in xenografts (n = 6/group). Scale bars: 50 μm. **M** Western blotting analyses showing that SOX2 protein expression was reduced in shSMYD3 group than in shNC group in vivo. *P < 0.05, **P ≤ 0.01, and ***P ≤ 0.001

To verify the results of above analyses, the basal expression of SMYD3 mRNA and protein were examined in CAL-27 and UM-SCC-1 cells (Additional file 1: Fig. S5A, B). SMYD3 was knocked down in both OSCC cell lines (Fig. 4D, Additional file 1: Fig. S5C). Consistently, SMYD3 suppression markedly impaired the ability to form tumorspheres and reduced OSCC cell proliferation (Fig. 4A–C, Additional file 1: Fig. S5D–G). These results were accompanied by a considerable decrease in pluripotency associated markers, including c-MYC, BMI1, NANOG, and SOX2 [25] expression (Fig. 4D). We also observed that knockdown of SMYD3 inhibited cellular H3K4me3 levels (Fig. 4D). Conversely, SMYD3 overexpression produced the opposite effects (Fig. 4E–H, Additional file 1: Fig. S5H–K). Notably, overexpression of an enzymatically deficient SMYD3 (EEL) did not alter the level of H3K4me3, in contrast to wild-type SMYD3 overexpression, indicating the dependence of H3K4me3 changes on SMYD3’s methylase activity (Additional file 1: Fig. S5L).

We also investigated whether SMYD3 could contribute to tumorigenicity in vivo. An OSCC cell line (CAL-27) stably expressing SMYD3 shRNA was constructed (Additional file 1: Fig. S5M, N), and the cells were injected subcutaneously into nude mice. As shown in Fig. 4I–K, tumors from OSCC cells in which SMYD3 was suppressed were considerably smaller compared with the control group. Furthermore, the tumors in the shSMYD3 group exhibited reduced proliferative activity as determined by Ki67 IHC staining and decreased self-renewal capacity indicated by SOX2 expression, compared to the control group (Fig. 4L, M). Taken together, we confirmed that SMYD3 could promote OSCC cell stemness maintenance and proliferation in vitro and tumorigenicity in vivo.

### Pharmacological inhibition of SMYD3 suppresses OSCC cell growth and impedes chemical-induced primary OSCC formation

**Fig. 5:**
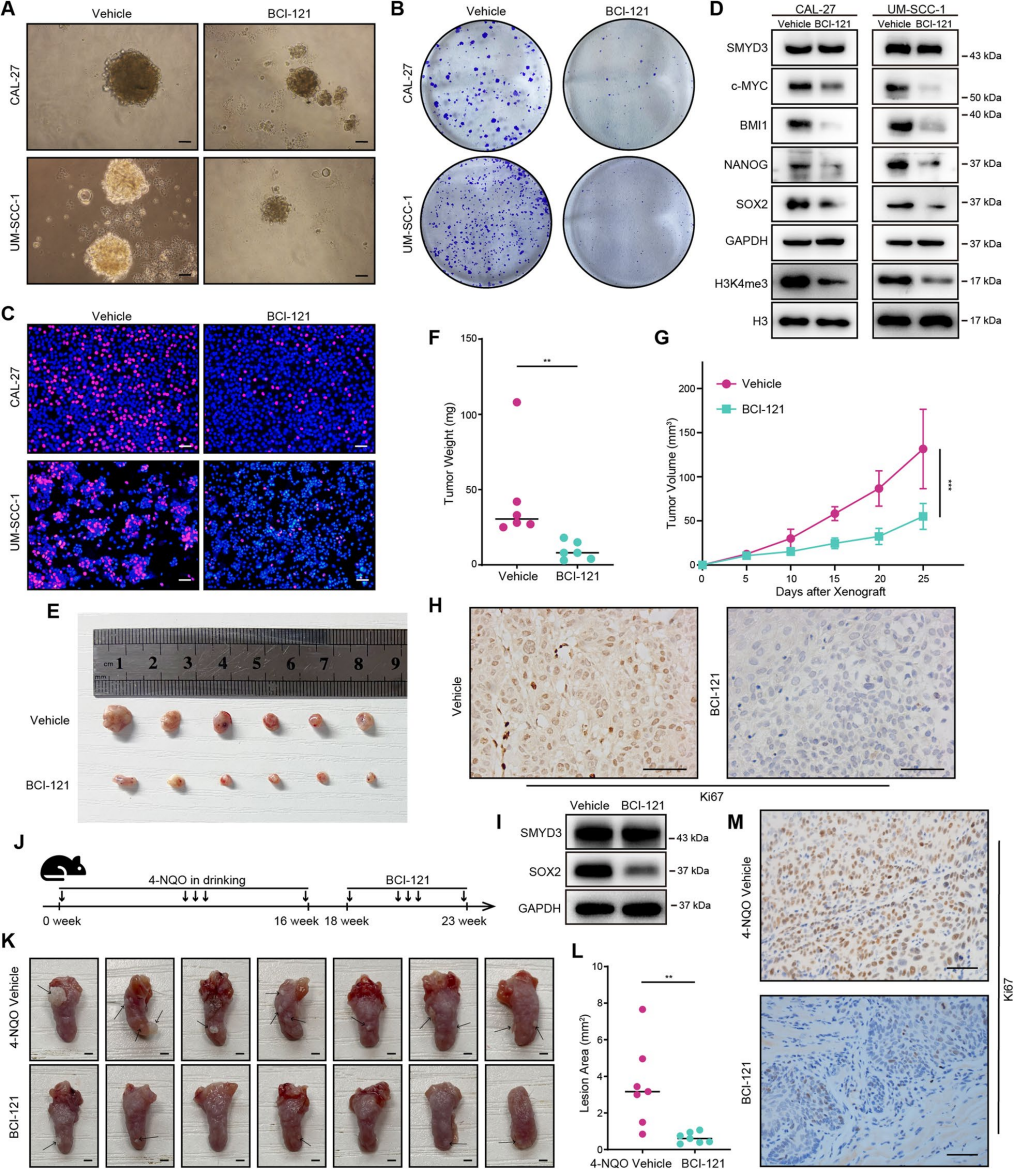
Pharmacological inhibition of SMYD3 suppresses OSCC cells growth and impedes the chemical-induced primary OSCC formation. **A** The ability of secondary tumorsphere formation was significantly reduced in BCI-121-treated OSCC cells relative to cells treated with vehicle. Representative images were shown. Scale bars: 50 μm. **B** The colony formation potential was inhibited following BCI-121 treatment as compared to vehicle treatment. **C** The ability of proliferation was suppressed in BCI-121-treated OSCC cells relative to cells treated with vehicle. Representative images were shown. Scale bars: 50 μm. **D** Western blotting analyses showing that c-MYC, BMI1, NANOG, SOX2 and H3K4me3 protein expression were decreased in BCI-121-treated CAL-27 and UM-SCC-1 cells. **E** Subcutaneous tumor formation in nude mice of BCI-121 treatment and vehicle groups (n = 6/group). **F**, **G** Tumor weight and tumor growth curves in the nude mouse xenograft model. **H** IHC staining for Ki67 in xenografts (n = 6/group). Scale bars: 50 μm. **I** Western blotting analyses showing that SOX2 protein expression was decreased in BCI-121 group than in vehicle group in vivo. **J** Experimental design of 4NQO-induced OSCC animal model and BCI-121 treatment. **K** Representative images of tongue lesions at 23 weeks after treatment (n = 7/group). Scale bars: 1.5 mm. **L** Quantification of lesion areas visible in the tongue from BCI-121 treatment and vehicle groups. **M** IHC staining for Ki67 in OSCC tissues (n = 7/group). Scale bars: 50 μm. *P < 0.05, **P ≤ 0.01, and ***P ≤ 0.001

To confirm the translational value of these findings, we assessed the effects of a SMYD3 selective inhibitor BCI-121 [21, 26, 27]. The level of H3K4me3 and cell viability varied in the presence of different dosages of BCI-121 treatment, and decreased notably when the concentration reached 200 µM and 350 µM in CAL-27 and UM-SCC-1, respectively. These two concentrations were used in subsequent experiments (Additional file 1: Fig. S6A–C). As expected, BCI-121 did not affect the level of SMYD3, but substantially repressed tumorsphere formation and OSCC cell proliferation in vitro (Fig. 5A–D, Additional file 1: Fig. S6D–H). The expressions of c-MYC, BMI1, NANOG, and SOX2 were also significantly decreased in the group that received BCI-121 (Fig. 5D). Moreover, intratumoral injection of BCI-121 effectively suppressed the tumor growth/proliferation and SOX2 expression of CAL-27 cells in vivo (Fig. 5E–I). We required 16–18 weeks to develop a 4-NQO-induced OSCC mouse model, which was followed by intraperitoneal administration of BCI-121 three times a week from the 18th to the 23rd week, to investigate the possibility of hindering OSCC tumorigenesis in vivo through this treatment (Fig. 5J). The untreated mice developed visible lesions on the tongue after 4-NQO induction (Fig. 5K). However, the lesion areas and Ki67 expression of tumors decreased significantly in mice treated with BCI-121, and histological alterations also were observed (Fig. 5L, M, Additional file 1: Fig. S7A, B). These results revealed that BCI-121 inhibited histone methyltransferase activity of SMYD3 and demonstrated therapeutic promise for the treatment of OSCC.

### HMGA2 is a downstream target gene of SMYD3

**Fig. 6:**
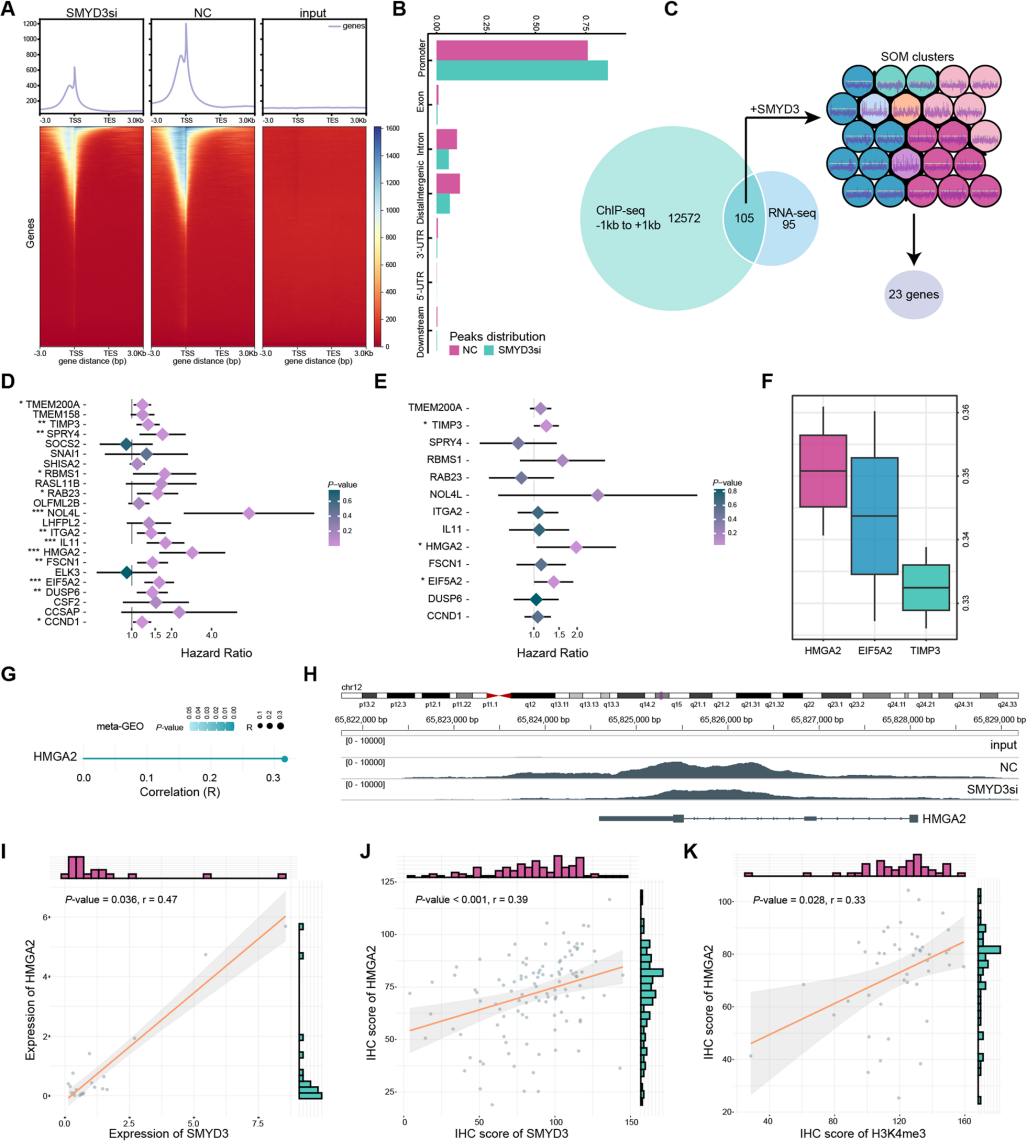
HMGA2 is a key downstream effector for SMYD3-mediated functions. **A**, **B** Binding peak signal distribution locations in ChIP-seq. **C** Flow chart for screening 23 downstream target genes. **D**, **E** Univariate and multivariate Cox regression analyses of the 23 genes in the meta-GEO dataset. Hazard Ratio and *P*-values were displayed. **F** The distributions of functional similarities of three proteins were summarized as boxplots. **G** SMYD3 was co-expressed with HMGA2 in the meta-GEO dataset. **H** The variation of H3K4me3 binding peaks was mainly at the promoter regions (− 1 kb to the TSS) of HMGA2. **I** Quantification of qRT-PCR of SMYD3 was correlated with that of HMGA2 in collected 20 OSCC samples. **J** Quantification of SMYD3 IHC staining was correlated with that of HMGA2 IHC staining in OSCC samples (n = 131, from Stomatological Hospital of Shandong University and Shanghai Qutdo Biotech Company). **K** Quantification of HMGA2 IHC staining was correlated with that of H3K4me3 IHC staining in OSCC samples (n = 45, from Shanghai Qutdo Biotech Company)

Based on the observations reported above, we identified critical genes regulated by SMYD3 through histone methyltransferase activity. We performed ChIP-seq to profile the differences in the genomic distribution of H3K4me3 in CAL-27 cells transfected with NC and SMYD3 siRNA. As shown in Fig. 6A, B, the overall presence of H3K4me3 throughout the genome decreased with SMYD3 knockdown. In addition, the binding peaks were significantly enriched in the transcription start site (TSS) region, primarily in the promoter region. We subsequently focused on the 12,677 genes whose binding peak centers covered − 1 kb to + 1 kb to the TSS. Significant signal differences were observed between the two groups, as they were more likely to be regulated at the transcriptional level (Additional file 2: Table S2). We identified 105 overlapping genes by comparing 200 downregulated genes (excluding SMYD3) identified from the RNA-seq with the decreased H3K4me3 occupancy genes (− 1 kb to + 1 kb) from the ChIP-seq (Fig. 6C, Additional file 2: Table S3). Then we performed the SOM algorithm on the 105 genes and SMYD3 in the meta-GEO dataset to search for highly correlated genes. The metric of within cluster sum of squares indicated that the ideal clustering size was seven, and 23 genes were found in the same module as SMYD3 (Fig. 6C, Additional file 1: Fig. S7C, Additional file 2: Table S3).

To evaluate the biological significance of these 23 genes, we carried out univariate and multivariate Cox regression analyses in meta-GEO dataset. The results indicated that HMGA2, TIMP3 and EIF5A2 could be deemed as independent prognostic factors for OSCC patients (Fig. 6D, E). In search of the vital downstream target of SMYD3, we measured the functional similarity among three proteins. HMGA2 was displayed the strongest relationship in biological process, function and component among the proteins in the interactome (Fig. 6F). As a proto-oncogenic chromatin regulator [28–30], HMGA2 promotes self-renewal and maintenance of cancer stem cell, and involves in different steps of tumorigenesis and malignant progression of HNSCC/OSCC [22, 23, 31–34]. However, the relationship between SMYD3 and HMGA2 was still unknown. In this study, we determined that SMYD3 was co-expressed with HMGA2 in OSCC (Fig. 6G). Results from the ChIP-seq revealed that H3K4me3 binding peaks were located at the upstream and downstream regions in the TSS of HMGA2, and the variation was significantly different between the NC and SMYD3 siRNA groups (Fig. 6H).

In meta-GEO dataset, HMGA2 was overexpressed in tumor samples compared with the normal samples, and patients with elevated HMGA2 expression had a worse prognosis than those with low HMGA2 expression (Additional file 1: Fig. S8A, B). In addition, we examined HMGA2 expression in samples collected with RT-qPCR, Western blotting, and IHC staining. The results confirmed that HMGA2 was upregulated in OSCC and associated with tumor malignancy (Additional file 1: Fig. S8C–E). Interestingly, there was a positive correlation between SMYD3 and HMGA2 at the transcriptional level and at the protein level (r = 0.47, *P* = 0.036; r = 0.39, *P* < 0.001; Fig. 6I, J, Fig. 2N, Additional file 1: Fig. S8E). Correspondingly, the IHC staining illustrated that HMGA2 expression was linked to the H3K4me3 level (r = 0.33, *P* = 0.028; Fig. 6K, Additional file 1: Fig. S3C–F).

**Fig. 7:**
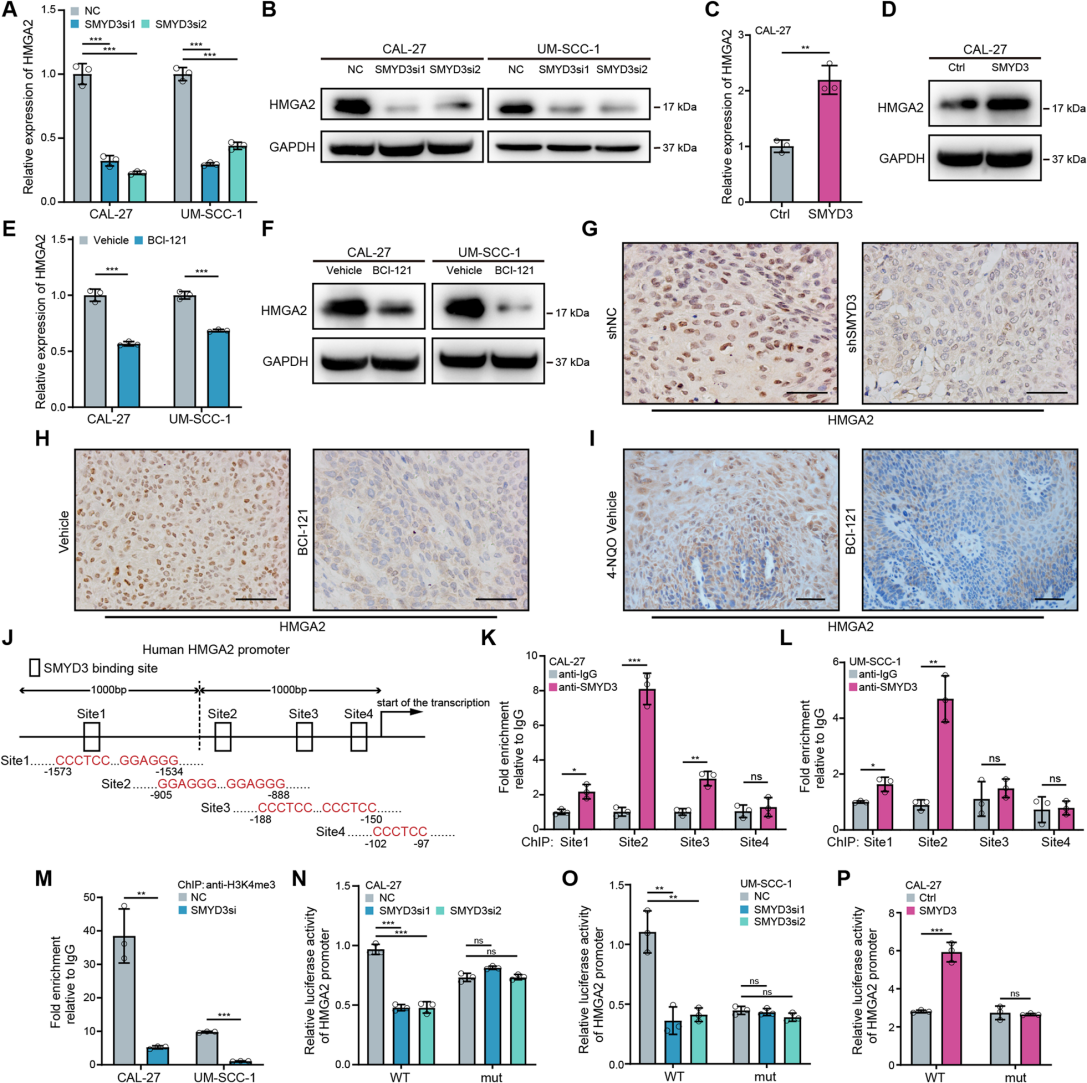
SMYD3 enhances HMGA2 transcription by binding to the HMGA2 promoter and increasing H3K4me3 modification. **A**, **B** The mRNA and protein levels of HMGA2 in CAL-27 and UM-SCC-1 cells transfected with SMYD3 siRNA. **C**, **D** The mRNA and protein levels of HMGA2 in CAL-27 and UM-SCC-1 cells transfected with SMYD3 plasmid. **E**, **F** The mRNA and protein levels of HMGA2 in CAL-27 and UM-SCC-1 cells following BCI-121 treatment. **G** HMGA2 expression was significantly decreased in the shSMYD3 group than in the shNC group of xenografts (n = 6/group). Scale bars: 50 μm. **H** HMGA2 expression was significantly reduced in the BCI-121 treatment group than in the vehicle group of xenografts (n = 6/group). Scale bars: 50 μm. **I** HMGA2 expression was significantly reduced in the BCI-121 treatment group than in the vehicle group of 4NQO-induced OSCC tissues (n = 7/group). Scale bars: 50 μm. **J** The SMYD3 potential binding sites in human HMGA2 promoter. **K**, **L** ChIP assays were performed to identify occupancy of the HMGA2 promoter in OSCC cells using SMYD3 antibodies. **M** Enrichment of H3K4me3 on the Site2 fragment of HMGA2 promoter. **N**–**P** Transcriptional activity of SMYD3 was assessed using a luciferase reporter system in OSCC cells. Ns, not significant, *P < 0.05, **P ≤ 0.01, and ***P ≤ 0.001

In vitro, knockdown of SMYD3 in CAL-27 and UM-SCC-1 cells significantly inhibited the HMGA2 transcription and protein levels (Fig. 7A, B). SMYD3 overexpression had the opposite effect (Fig. 7C, D). Similarly, the addition of BCI-121 reduced HMGA2 expression (Fig. 7E, F). Not surprisingly, HMGA2 expression was significantly decreased in the shSMYD3 and BCI-121 groups compared with the control group in vivo (Fig. 7G–I). Thus, our data indicated that SMYD3 regulated the expression of HMGA2, which may be involved in OSCC tumorigenesis.

### SMYD3 enhances HMGA2 transcription by binding to the HMGA2 promoter and increasing H3K4me3 modification

We subsequently explored the mechanisms by which SMYD3 upregulated HMGA2 expression. Based on existing studies and our ChIP-seq results, we hypothesized that SMYD3 could bind to the 5′-CCCTCC-3′ or 5′-GGAGGG-3′ motifs in the gene promoter and exert histone methyltransferase activity through the SET domain to regulate gene transcription in OSCC [15, 35]. Four binding sites containing 5′-CCCTCC-3′ and 5′-GGAGGG-3′ within − 2 kb to the TSS of the HMGA2 genome were identified (Fig. 7J). Our attention in the ChIP-qPCR findings was directed towards identifying the most conserved regions. We discovered that while SMYD3 bound to both Site1 and Site2 regions in CAL-27 and UM-SCC-1 cells, it exhibited the highest affinity for the Site2 region amongst the four locations adjacent to the TSS of HMGA2. As a result, we have decided to conduct further investigation on the Site2 region (Fig. 7K, L). Remarkably, SMYD3 knockdown markedly reduced the level of H3K4me3 on the Site2 at the proximal promoter for HMGA2 (Fig. 7M). We constructed luciferase reporter plasmids containing the HMGA2 promoter region (WT, in which the Site2 was intact) and HMGA2 mutant promoter region (mut, in which the Site2 was mutated) (Additional file 1: Fig. S9A). As expected, SMYD3 knockdown decreased HMGA2 promoter-based luciferase activity (Fig. 7N, O), and SMYD3 overexpression displayed the opposite effect (Fig. 7P). We performed rescue experiments by co-transfecting HMGA2 siRNA with a SMYD3-overexpressing plasmid and SMYD3 siRNA with a HMGA2-overexpressing plasmid in CAL-27 cells (Additional file 1: Fig. S9B, C, Fig. S10A, B). The SOX2 expression, tumorsphere formation and proliferation capacities of SMYD3/HMGA2-upregulated cells were nullified by HMGA2/SMYD3 knockdown (Additional file 1: Fig. S9D–H, Fig. S10C–G). These findings further suggested that HMGA2 was the principal effector of SMYD3-mediated functions.

**Fig. 8:**
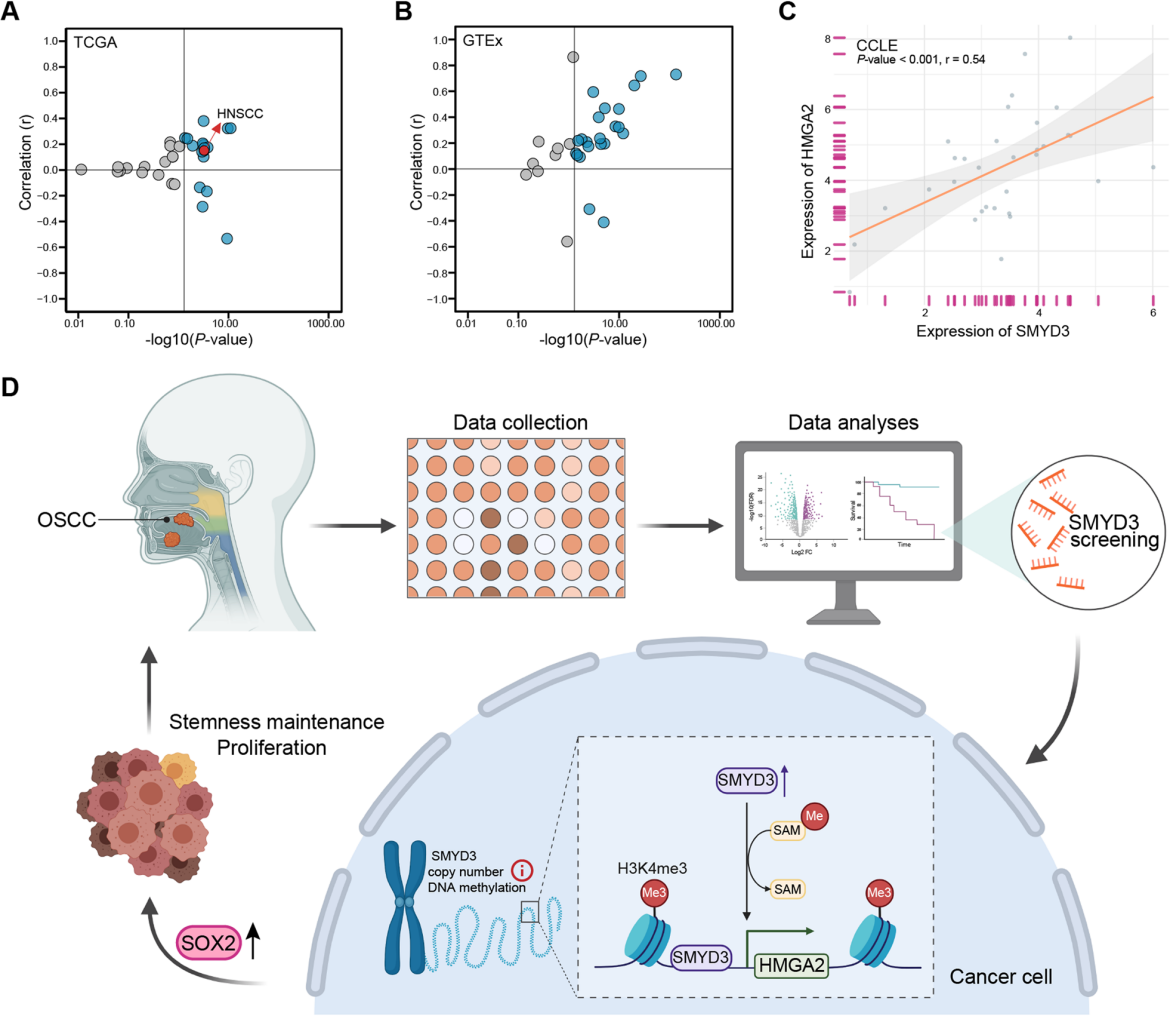
The regulatory association of SMYD3 with HMGA2 could be universal. **A** In the TCGA pan-cancer dataset, SMYD3 was positively correlated with HMGA2 expression in most tumor tissues. **B** In the GTEx database, SMYD3 was positively correlated with HMGA2 expression in most normal tissues. **C** In the CCLE database, SMYD3 was positively correlated with HMGA2 expression in most upper respiratory gastrointestinal tract tumor cell lines. **D** Graphical abstract for SMYD3 promoting tumorigenesis of OSCC

We downloaded and processed the gene expression data for 33 tumor tissues (the pan-cancer level) from the TCGA database, 31 normal human tissues from the GTEx database, and 32 upper respiratory and gastrointestinal tract tumor cell lines from the CCLE database. It was revealed that SMYD3 was positively correlated with HMGA2 expression in most tumor and normal tissues and cell lines (Fig. 8A–C). Therefore, the regulatory effects of SMYD3 on HMGA2 appeared to be ubiquitous in a wide range of tissues and cells.

## Discussion

Oral squamous cell carcinoma (OSCC) is a heterogeneous tumor originating from the lining epithelium of the oral mucosa and possesses unique pathological features [36]. Epigenetic mechanisms have robust effects on OSCC tumorigenesis [37]. Most epigenetic modifications occur in nucleus and are governed by specific modifying enzymes at the DNA, RNA, histone, or chromatin levels, thereby globally regulating gene expression [37, 38]. In this study, we focused on chromatin regulators that modulate local or global epigenetic patterns and discovered that some were deregulated in OSCC. Additional screening revealed that SMYD3 was robustly upregulated in OSCC tissues, especially cancer cells, and the ROC curves showed promising results. Moreover, the expression of SMYD3 differed among OSCC subtypes. In SMYD3-enriched subtypes, including classical, TP-53 mutation, smoking and alcohol consumption groups, patients tended to exhibit unfavourable tumor differentiation and prognosis [39]. Survival analysis revealed that excessive SMYD3 expression also correlated with a poor prognosis in OSCC. Therefore, SMYD3 could be a potential diagnostic and prognostic biomarker for OSCC. Still, large number of prospectively enrolled patients is warranted to confirm the prognostic utility of SMYD3 in OSCC and a cut-off value of SMYD3 expression should be unified before its translation into the clinic. In addition to SMYD3, first screening in the TCGA and meta-GEO datasets detected SETMAR as a downregulated gene and a protective prognostic factor in OSCC. SETMAR has been reported to be a protein lysine methyltransferase that mediates methylation of H3K27 and H3K36, and dysregulation of SETMAR has been associated with several cancers [40]. Our results suggested that SETMAR might be a tumor suppressor gene in OSCC. Considering tumorigenesis is a synergetic process with a broad involvement of oncogenes and tumor suppressor genes, the role of SETMAR and the relationship between SMYD3 and SETMAR in OSCC deserve further investigation.

A recent report indicated that gene locus amplification resulted in SMYD3 upregulation in hepatocellular carcinoma [41]. In this study, we confirmed that the frequency of SMYD3 genomic amplification was increased, and the copy number was significantly correlated with mRNA levels in OSCC. Previous studies reported that DNA methylation levels are dysregulated in most cancers [42], and the methylation level in the SMYD3 promoter region is low in colorectal cancer [43]. In the TCGA database, OSCC patients were classified into four subtypes based on DNA methylation levels, and SMYD3 expression varied significantly among these four groups. We also found SMYD3 expression was closely related to its DNA methylation status. These results suggest that CNV and DNA hypomethylation might contribute to SMYD3 overexpression in OSCC. Moreover, it has been appreciated that non-coding RNA can regulate the expression of SMYD3 [17]. With regard to the mechanisms responsible for aberrant SMYD3 expression in cancers, several mechanistic models are needed to be proposed at diverse regulatory levels in the future.

SMYD3 is considered as an essential regulator of cancer stem cell characteristics [44, 45]. In this study, we identified five clusters and underlying differentiation trajectories in epithelial cells through single-cell RNA-seq analyses. The C5 group expressing high level of SMYD3 possesses strong stemness properties, which indicates SMYD3 may be considered as a potential biomarker for cell stemness in OSCC. Furthermore, we verified that OSCC tumorigenicity and proliferation were significantly inhibited or enhanced when SMYD3 was disrupted or overexpressed in the cells. This was confirmed by changes in the expression of cell stemness markers, including c-MYC, BMI1, NANOG, and SOX2 [46, 47]. Multiple cellular biological functions including proliferation, metastasis, and tumor microenvironment remodelling are closely related to cancer cell stemness [48]. Henceforth, further experiments, encompassing knockout mouse models and self-renewal phenotypes in vivo, are requisite to substantiate the correlation between SMYD3 and cell stemness in OSCC.

H3K4 methylation is recognized as a hallmark event associated with gene transcription [49]. SMYD3 was initially defined as a H3K4 methyltransferase to promote H3K4me3 formation and activate downstream gene transcription to participate in cancer development. SMYD3 has also been reported to directly bind to the 5′-CCCTCC-3′ and 5′-GGAGGG-3′ motifs that are specific to the promoter region of genes [15]. For example, SMYD3 binds to the hTERT promoter region and increases H3K4me3 levels, activating hTERT in trans [50]. Of particular interest, Sarris et al. [7] demonstrated in their model that SMYD3-bound sequences are not enriched in the 5′-CCCTCC-3′ or 5′-GGAGGG-3′ motifs. The findings of this study revealed that SMYD3 facilitated cell stemness maintenance, cell proliferation, and tumorigenesis of OSCC by specifically binding to the 5′-GGAGGG-3′ motifs present in the promoter region, increasing H3K4me3 level, and activating HMGA2 transcription. HMGA2, which is known to promote tumor growth through various mechanisms, is found to be highly expressed in the majority of human malignancies, including HNSCC/OSCC. The detection of HMGA2 expression can serve as a valuable diagnostic and prognostic tool in the clinical management of HNSCC/OSCC [30, 32, 51–54]. Additionally, HMGA2 is regarded as a marker of cancer stem cells, and has the capability to initiate the formation of a colony in OSCC [55, 56]. A study has indicated that in OSCC, the LIN28B-Let7 axis enhances tumor cell stemness and promotes tumorigenesis by mediating SOX2 expression through HMGA2 [22]. Furthermore, the HMGA2-Snai2 axis can regulate the stemness and tumorigenicity of HNSCC [23]. Most studies on the regulation of HMGA2 expression have focused on post-transcription activity, primarily by non-coding RNAs [57]. This study is the first demonstration of the effects of histone methylation modifications of SMYD3 on HMGA2 expression in OSCC. In addition, the regulatory association of SMYD3 with HMGA2 could be generalized to a pan-cancer level. However, it is unlikely that SMYD3 is the sole histone methyltransferase responsible for the H3K4me3 modification at the HMGA2 chromatin, as our findings revealed that some tumors and tissues displayed a low correlation between SMYD3 and HMGA2 expressions. Also, it is imperative to discover additional specific DNA binding sites, including those located downstream of the TSS. Moreover, this study confirmed the biological significance of BCI-121, an inhibitor of SMYD3 enzyme activity [21], both in vitro and in vivo, particularly in the 4-NQO model of oral carcinogenesis. Therefore, our data supported the premise that the SMYD3 inhibitor is a potential therapeutic strategy for OSCC. The clinically relevant concentrations and doses are currently being explored, and therapeutic regimens targeting SMYD3 alone or in combination are also under investigation.

In addition to H3K4, other histone including H4K20 and H4K5, and non-histone substrates of SMYD3 have been revealed, mainly in the context of tumors. Specifically, Van Aller et al. demonstrated that SMYD3 primarily mono-methylates H4K5 rather than H3K4 and H4K20 [58]. In lung and pancreatic cancers, SMYD3 is localized in the cytoplasm, where it enhances RAS/ERK signaling by mediating the methylation of MAP3K2 kinase and preventing its interaction with the PP2A phosphatase complex [59]. The latest research suggested that SMYD3-mediated methylation of RNF113A impedes small cell lung cancer sensitivity to DNA alkylation damage [60]. Furthermore, a different study indicated that SMYD3 knockdown induced transcriptional upregulation of CD8 + T-cell attracting chemokines and APM components in HNSCC cells, also showing SMYD3 might mediate tumor progression and immune escape [61]. Thus, additional spatio-temporal studies at the single-cell level and exploring other regulatory mechanisms of SMYD3 in OSCC warrant consideration.

## Conclusions

This study identified an oncogenic role for SMYD3 in OSCC and revealed an epigenetic regulatory mechanism of SMYD3 at the HMGA2 locus (Fig. 8D). Moreover, SMYD3 demonstrated potential as a diagnostic and prognostic indicator for OSCC, and its chemical inhibitor, BCI-121, inhibited the growth and proliferation of OSCC. Taken together, these results suggest that SMYD3 could serve as a promising biomarker and therapeutic target for OSCC.

## Materials and methods

### Clinical specimen collection

We collected samples of pathologically diagnosed OSCC from Stomatological Hospital of Shandong University and Shanghai Qutdo Biotech Company with written informed consent and approval from Shandong University Research Ethics Committee and Shanghai Qutdo Biotech Company Ethics Committee. Totally, 32 pairs of fresh tumor and adjacent normal tissues, and 131 paraffin-embedded specimens were collected (Additional file 2: Table S4).

### Publicly accessible data acquisition, processing and differentially expressed gene (DEG) analysis

Microarray gene expression and clinical annotation data from GSE9844, GSE30784, GSE41613, GSE42743, GSE74530, GSE78060, GSE138206, GSE37991 and GSE146483 of the Gene-Expression Omnibus (GEO) database were downloaded. In the GSE30784 dataset, where gene expressions of 167 OSCC, 17 oral dysplasia, and 45 normal oral tissues were compared, a better reflection of the relationship between genes and tumorigenesis could be obtained [62]. We obtained RNA sequencing (RNA-seq), CNV, DNA methylation and clinical characteristic data from The Cancer Genome Atlas (TCGA) database, which comprised of 281 OSCC and 29 oral normal samples. The data was acquired through the GDC portal, cBioPortal atlas and MEXPRESS website with a strict adherence to the integrity of clinicopathologic features and anatomic sites as outlined in Additional file 2: Table S5. We processed the data as previously reported [2] and leveraged the “removeBatchEffect” function of the R package “limma” to merge the data from the same microarray platform to yield a meta-GEO dataset (n = 402/OSCC group, n = 108/normal group). Gene expression data (FPKM value) of 40 Asian OSCC samples and clinical characteristic data were downloaded from the International Cancer Genome Consortium (ICGC) database. And we obtained gene expression data (TPM value) of TCGA pan-cancer, 32 upper respiratory gastrointestinal tract cancer cell lines and 31 human normal tissue from the UCSC Xena, Cancer Cell Line Encyclopedia (CCLE) and Genotype-Tissue Expression (GTEx) database respectively. For single-cell RNA-seq data, GSE103322 [24] was mined by “Scanpy”. We ran “Harmony” to eliminate the batch effects among cells. Uniform Manifold Approximation and Projection (UMAP) reduction was used for cluster visualization, and R package “Monocle2” was performed to arrange cells into a trajectory which was divided into different branches to imitate cell evolution or differentiation. The DEGs between OSCC and oral normal samples in the TCGA database were analysed by means of the R package “edgeR” when the criteria fold change > 2 and false discovery rate (FDR) < 0.05 were met.

### Feature selection for OSCC

Lasso logistic regression is a machine learning that identifies variables by choosing outputs with the lowest classification error [63]. Random forest based on the Boruta algorithm can extract features and calculate the importance [64]. Here, the R package “glmnet” and “Boruta” were used to screen for DEGs closely associated with OSCC tumorigenesis.

### Biological function and pathway enrichment analysis, and semantic similarity measurement

Scoring analysis of gene sets was performed with the scanpy.tl.score_genes function and gene set variation analysis (GSVA) in the single-cell RNA-seq dataset. Single-sample gene set enrichment analysis (ssGSEA) was leveraged to quantify the activity of cancer cell stemness and proliferation in the meta-GEO dataset. Gene ontology (GO) and Kyoto encyclopaedia of genes and genomes (KEGG) pathway analysis via KOBAS 3.0 [65] were conducted to measure the enriched biological processes and pathways for genes. The cut-off criterion was corrected *P* < 0.05. And gene set enrichment analysis (GSEA) was applied to verify the results of GO and KEGG analysis. The cut-off criteria for GSEA were nominal *P* < 0.05 and FDR < 0.25. The gene sets used in this study were downloaded from MSigDB (https://www.gsea-msigdb.org/gsea/msigdb/index.jsp) and CancerSEA (http://biocc.hrbmu.edu.cn/CancerSEA/) databases. Based on the semantic similarities of GO terms (biological process, molecular function and cellular component) used for gene annotation, we ranked the potential downstream target genes of SMYD3 by average functional similarities between the protein and its interaction partners [66]. Semantic similarities among interactome proteins were measured through the R package “GOSemSim”.

### Western blotting and immunohistochemistry (IHC) staining

Details of protein extraction, Western blotting, IHC staining and IHC score obtainment referred to our previous studies [2]. Primary antibodies against GAPDH (ab9458, Abcam, UK), SMYD3 (ab187149, Abcam, UK), HMGA2 (ab97276, Abcam, UK), NANOG (ab109250, Abcam, UK), c-MYC (ab32072, Abcam, UK), BMI1 (ab126783, Abcam, UK), Histone H3 (ab1791, Abcam, UK), SOX2 (ab92494, Abcam, UK), H3K4me3 (ab8580, Abcam, UK) and Ki67 (ab15580, Abcam, UK) were utilized.

### Cell culture, transient transfection, RNA extraction and quantitative real-time PCR (qRT-PCR)

Human OSCC cell lines (CAL-27 and UM-SCC-1) were used in this research, and the source and culture conditions were the same as in previous study [5]. Polyplus (PolyPlus-transfection, France) was utilized to transfect Negative Control (NC), SMYD3 siRNAs (RiboBio, Guangzhou, China) and HMGA2 siRNAs (RiboBio, Guangzhou, China) into OSCC cells according to the manufacturer’s protocol, and cells were collected after 48–72 h transfection. The virus (containing short hairpin SMYD3 (shSMYD3) or shNC, Genechem, Shanghai, China) were transduced into OSCC cells with a multiplicity of infection of 100, as recommended. Roche Transfection Reagent (Roche, Switzerland) was utilized for the transfection of SMYD3, histone methyltransferase-inactive mutant-SMYD3 (EEL) [15], and HMGA2 plasmids (Weizhenbio, Jinan, China) according to the provided protocol, and cells were collected after 48 h transfection. The procedures of RNA extraction and qRT-PCR were as shown before [2]. The sequences of siRNA/shRNA and the primers used in this experiment are listed in Additional file 2: Table S6.

### Tumorsphere formation, colony formation, 5-ethynyl-2’-deoxyuridine (EdU) staining, and cell viability assays

For tumorsphere formation assay, the disassociated single cells (CAL-27: 4000–6000 cells/well; UM-SCC-1: 10,000 cells/well) were cultured in serum-free DMEM/RPMI-1640 supplemented with B27 (Life Technologies, Invitrogen, US), EGF (Sigma-Aldrich, US), bFGF (Sigma-Aldrich, US), insulin (Life Technologies, Invitrogen, US) and bovine serum albumin (Sigma-Aldrich, US), and grown in a 12-well ultra-low-attachment plate (Corning, US) for 5–7 days. These tumorspheres were harvested and dissociated, followed by re-plating to form secondary sphere in aforementioned media for another 5–7 days. Tumorspheres with diameter larger than 50 μm were counted. For colony formation assay, the cells (1000 cells/well) were seeded in a 6-well plate and incubated for 1–2 weeks until colonies of cells appeared. For EdU staining, the fraction of DNA-replicating cells was assessed according to the manufacturer’s instruction (C10310-3, RiboBio, Guangzhou, China). Cell viability was assayed using the CCK-8 reagent (HY-K0301, MedChemExpress) as previously described in our research [67].

### Tumor xenograft and 4-nitroquinoline 1-oxide (4-NQO) mouse model development

We used 24 thymus-null BALB/c nude mice (male, 5–6 weeks old) purchased from Vital River Labs (Beijing, China) for the tumor xenograft construction. For the subcutaneous injection model, four groups (n = 6/group) were randomly divided. CAL-27 cells (3 × 10^6^ cells) transduced with/without SMYD3 shRNA or shNC were injected subcutaneously into the right flank of nude mice. After 5 days of incubation, 50 µl of BCI-121 (1000 µM, Selleck, US) and vehicle control prepared according to the manufacturer’s instruction were administrated intratumorally into two groups three times a week [41]. Tumor growth was examined every 5 days, and the mice were euthanized 25 days after injection. Proteins were extracted from the tumor xenografts for further experiments. Fourteen C57BL/6 mice (male, 6–8 weeks old) were purchased from Vital River Labs (Beijing, China) for the 4-NQO (Santa Cruz Biotechnology, US) mouse model construction. Tongue cancer was induced by 4-NQO (100 μg/mL) in the drinking water for 16 weeks. Mice were randomly divided into 2 groups (n = 7/group) at week 18 and intraperitoneally given vehicle control and BCI-121 (50 mg/kg) respectively three times a week for 5 weeks. At the end of week 23, the mice were sacrificed to obtain the lesions in the tongue [68–70]. Paraffin-embedded tongue tissues were then sliced for hematoxylin and eosin (H&E) staining. All animal experiments were approved by Shandong University Research Ethics Committee of the School of Basic Medical Sciences.

### RNA-seq, chromatin immunoprecipitation (ChIP)-seq, and ChIP assay

Two groups of CAL-27 transfected with NC and SMYD3 siRNA were performed poly-A RNA-seq by RiboBio (Guangzhou, China) and ChIP-seq by Xiuyue Biol (Jinan, China). The detailed processing and analysis criteria of RNA-seq were referred to our previous study [71], and the count data was shown in Additional file 2: Table S7. For the ChIP-seq, we completed the procedure as previously reported [72], and Drosophila cell chromatin (as spike-in control) was added during processes to eliminate the effect of transfection on histone methylation at the overall cellular level [73]. The antibody utilized was an anti-H3K4me3 (39,159, ActiveMotif, US). ChIP assay was conducted using the SimpleChIP^®^ Enzymatic Chromatin IP Kit (56,383, Cell Signaling Technology, US) according to the manufacturer’s protocol. The Chromatin supernatants were incubated with antibodies including an anti-SMYD3 (ab228015, abcam, US) and anti-H3K4me3 (ab8580, abcam, US).

### Dual luciferase reporter gene assay

Human HMGA2 wild-type (WT) promoter fragment in pGL3-basic luciferase plasmid were synthesized by GENEray Biotechnology (Shanghai, China). The SMYD3 binding sites with the sequence GGAGGG were mutated to TTTTTT using the KOD-PlusMutagenesis Kit (TOYOBO, Japan) and the resulting vector was named pGL3-SMYD3/mutant (mut). The Luciferase Assay System (Promega, Madison, WI, USA) was leveraged to measure the luciferase reporter activity.

### Self-organizing map (SOM) network construction

The R package “kohonen” was utilized to import the gene expression of tumor samples of the meta-GEO dataset into “SOM”. SOM is a data matrix and visualization technique based on neural networks. It involves finding a set of center points (also known as codebook vectors) and then mapping each object in the dataset to the corresponding center point based on the most similar principle. In SOM, there exists a topological order between the center points, such that when one center point is updated, its neighboring center points are also updated until a certain threshold is reached or the center points no longer undergo significant changes. Ultimately, a series of center points (codes) are obtained, which implicitly define multiple clusters, where the objects closest to a particular center point are assigned to the same cluster. In SOM clustering, genes assigned to the same module exhibit similar expression patterns [74]. By performing N iterations and convergence, we obtained distinct modules in which high similarity of gene expression within the same module, and delved out the genes in the same module with SMYD3. These genes could be the downstream targets that might be regulated by SMYD3.

### Statistical analysis

To filter the chromatin regulators with the paramount prognostic significance, we used the random forest algorithm in the R package “randomForestSRC” to rank the variables according to their importance to overall survival (OS). Analyses of OS and progress-free survival (PFS) were performed by Kaplan–Meier analyses and log-rank tests, and the cut-off point of each dataset subgroup was calculated using the R package “survminer”. In addition, Cox proportional hazard regression models were constructed using univariate and multivariate analyses to determine the independent prognostic factors. For comparisons of two groups, statistical significance for normally and non-normally distributed variables was evaluated by unpaired Student's t-tests and Mann–Whitney U tests, respectively. For comparisons of more than two groups, one-way ANOVA tests or Kruskal–Wallis tests were utilized. Pearson’s or Spearman’s correlation analysis were leveraged to measure the linear relationship between two groups. The R package “RCircos” was used to plot the CNV landscape of SMYD3. The receiver operating characteristic (ROC) curve analysis was performed to evaluate the sensitivity and specificity of SMYD3 expression for OSCC diagnosis. All statistical analyses were performed using R software (version 4.1.2). Each experiment was repeated three times or more, and data are illustrated as mean ± standard deviation. Statistical significance was depicted as follows: ns, not significant; *P < 0.05; **P ≤ 0.01; ***P ≤ 0.001.

## Supplementary Information

**Additional file 1: Fig. S1.** Identification of SMYD3 for diagnosis of OSCC. **A**–**E** ROC curve analyses of SMYD3 in TCGA, meta-GEO, TCGA, GSE37991, and GSE30784 datasets. AUC values are shown. **Fig. S2.** The DNA methylation and genomic mutation profile in the TCGA-OSCC dataset. **A** The correlation of SMYD3 expression and DNA methylation level in TCGA-OSCC cohort. **B** Value differences of DNA methylation probes in normaland tumortissues from TCGA-OSCC cohort. **C** The lollipop plot illustrates the differential distribution of somatic mutation in the TCGA-OSCC dataset for SMYD3. **D**, **E** ROC curve analyses of SMYD3 in qRT-PCR and IHC staining of collected samples, respectively. Ns, not significant, *P < 0.05, **P ≤ 0.01, and ***P ≤ 0.001. **Fig. S3.** High expression of SMYD3 indicates increased H3K4me3 modification and HMGA2 expression. **A**–**F** IHC images of high and low protein expression of SMYD3, H3K4me3 and HMGA2. Scale bars: 100 μm. **Fig. S4.** Biological function and pathway enrichment analysis. **A** The results of GO analysis of RNA-seq on two groups of CAL-27 transfected with NC and SMYD3 siRNA. **B** The results of KEGG analysis of RNA-seq on two groups of CAL-27 transfected with NC and SMYD3 siRNA. **Fig. S5.** SMYD3 facilitates OSCC cell stemness maintenance and proliferation in vitro and tumorigenesis in vivo. **A**, **B** SMYD3 mRNA and protein levels in CAL-27 and UM-SCC-1 cell lines. **C** SMYD3 mRNA levels in OSCC cells transfected with NC and SMYD3 siRNAs. **D**–**G** Quantitative statistical results of SMYD3 knockdown in vitro experiments. **H** SMYD3 mRNA levels in OSCC cells transfected with vector and SMYD3 plasmid. **I**–**K** Quantitative statistical results of SMYD3 overexpression in vitro experiments.** L** The protein expressions of SMYD3 and H3K4me3 were detected after transfection of CAL-27 cell line with SMYD3 plasmids. **M**, **N** SMYD3 mRNA and protein levels in CAL-27 transfected with shNC and shSMYD3. *P < 0.05, **P ≤ 0.01, and ***P ≤ 0.001. **Fig. S6.** BCI-121 suppresses OSCC cells stemness maintenance and proliferation. **A** H3K4me3 levels in OSCC cells under different concentrations of BCI-121. **B**, **C** The effect of different concentrations of BCI-121 on cell viability determined by CCK8 assays in CAL-27 and UM-SCC-1 cell lines. **D** SMYD3 mRNA levels in OSCC cells following BCI-121 treatment. **E**–**H** Quantitative statistical results of BCI-121 treatment in vitro experiments. Ns, not significant, *P < 0.05, **P ≤ 0.01, and ***P ≤ 0.001. **Fig. S7.** BCI-121 impedes the chemical-induced primary OSCC formation. **A**, **B** Representative H&E images of tongue lesions. Scale bars, 200 μm. **C** The number of clusters was determined using SOM clustering. **Fig. S8.** HMGA2 is upregulated in OSCC, and high expression of HMGA2 predicts a poor prognosis. **A** HMGA2 was overexpressed in OSCC samples from the meta-GEO dataset. Numbers in parentheses indicate the sample size. **B** Patients with high HMGA2 expression have a worse prognosis than those with low HMGA2 expression in the meta-GEO dataset. Numbers in parentheses indicate the sample size. **C** Quantitative result of qRT-PCR of HMGA2 in 20 paired adjacent normal and OSCC tissues. **D** The protein levels of HMGA2 in 12 pairs of OSCC tissuesand adjacent normal tissuesmeasured by Western blotting.** E** Images of IHC staining for HMGA2 in normal tissues and different histologic grades of OSCC tissues. Scale bars: 50 μm. **Fig. S9.** HMGA2 is a downstream target gene of SMYD3. **A** The SMYD3 binding sitein human HMGA2 promoter and the corresponding base mutation. **B**, **C** HMGA2 mRNA and protein levels in CAL-27 transfected with NC and HMGA2 siRNA. **D** Upon the completion of the ten-day tumorsphere formation assay, the proteins were extracted from the spheroid cells on the 10th day. The expression of SOX2 in SMYD3-upregulated cells was nullified following HMGA2 knockdown.** E**–**H** The tumorsphere formation and proliferation capacities of SMYD3-upregulated cells were abrogated by HMGA2 knockdown. Scale bars: 50 μm. *P < 0.05, **P ≤ 0.01, and ***P ≤ 0.001. F**ig. S10.** SMYD3 affects OSCC cell stemness maintenance and proliferation via HMGA2. **A**, **B** HMGA2 mRNA and protein levels in CAL-27 transfected with vector and HMGA2 plasmid. **C**–**G** The SOX2 expression, tumorsphere formation and proliferation capacities of HMGA2-upregulated cells were abrogated by SMYD3 knockdown. Scale bars: 50 μm. *P < 0.05, **P ≤ 0.01, and ***P ≤ 0.001

**Additional file 2: Table S1**. The results of differential expression analysis on RNA-seq data.** Table S2**. The results of ChIP-seq.** Table S3**. The 105-overlapping-gene and 23-gene lists.** Table S4**. Clinicopathological characteristics of OSCC tissues and microarray chip.** Table S5**. Clinicopathological characteristics of OSCC samples in TCGA database.** Table S6**. The sequences of siRNA/shRNA and the primers for qRT-PCR.** Table S7**. The count data of RNA-seq.

## Data Availability

The published article includes all data sets generated/analysed for this study.

## Abbreviations

OSCC: Oral squamous cell carcinoma
SMYD3: SET and MYND domain-containing protein 3
HNSCC: Head and neck squamous cell carcinoma
HMGA2: High mobility group AT-hook 2
H3K4me3: Tri-methylation of histone 3 lysine-4
CNV: Copy number variation
TCGA: The Cancer Genome Atlas
GEO: Gene-Expression Omnibus
ICGC: International Cancer Genome Consortium
CCLE: Cancer Cell Line Encyclopedia
GTEx: Genotype-Tissue Expression
GSVA: Gene set variation analysis
GSEA: Gene set enrichment analysis
IHC: Immunohistochemistry
4-NQO: 4-Nitroquinoline 1-oxide
ChIP: Chromatin immunoprecipitation
SOM: Self-organizing map
OS: Overall survival
PFS: Progress-free survival
TSS: Transcription start site
