# Supplementary figures and images for: Histone lysine methyltransferase SMYD3 promotes oral squamous cell carcinoma tumorigenesis via H3K4me3-mediated HMGA2 transcription
Source: Clin Epigenetics. 2023 May 26;15:92. doi: 10.1186/s13148-023-01506-9 (PMC10223939; doi:10.1186/s13148-023-01506-9)

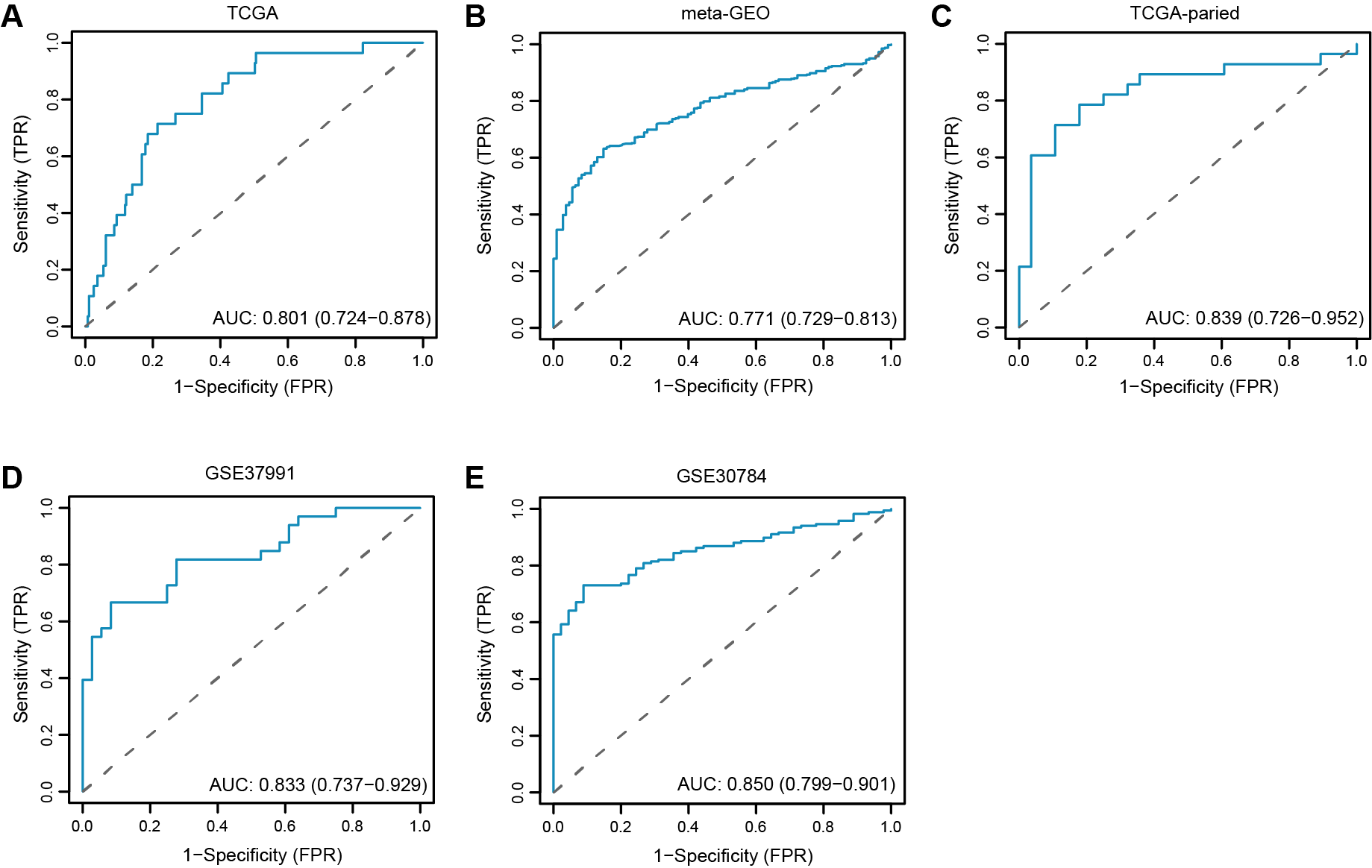

Supplement: Supplementary file 1 — Additional file 1: Fig. S1. Identification of SMYD3 for diagnosis of OSCC. A–E ROC curve analyses of SMYD3 in TCGA, meta-GEO, TCGA, GSE37991, and GSE30784 datasets. AUC values are shown. Fig. S2. The DNA methylation and genomic mutation profile in the TCGA-OSCC dataset. A The correlation of SMYD3 expression and DNA methylation level in TCGA-OSCC cohort. B Value differences of DNA methylation probes in normaland tumortissues from TCGA-OSCC cohort. C The lollipop plot illustrates the differential distribution of somatic mutation in the TCGA-OSCC dataset for SMYD3. D, E ROC curve analyses of SMYD3 in qRT-PCR and IHC staining of collected samples, respectively. Ns, not significant, *P < 0.05, **P ≤ 0.01, and ***P ≤ 0.001. Fig. S3. High expression of SMYD3 indicates increased H3K4me3 modification and HMGA2 expression. A–F IHC images of high and low protein expression of SMYD3, H3K4me3 and HMGA2. Scale bars: 100 μm. Fig. S4. Biological function and pathway enrichment analysis. A The results of GO analysis of RNA-seq on two groups of CAL-27 transfected with NC and SMYD3 siRNA. B The results of KEGG analysis of RNA-seq on two groups of CAL-27 transfected with NC and SMYD3 siRNA. Fig. S5. SMYD3 facilitates OSCC cell stemness maintenance and proliferation in vitro and tumorigenesis in vivo. A, B SMYD3 mRNA and protein levels in CAL-27 and UM-SCC-1 cell lines. C SMYD3 mRNA levels in OSCC cells transfected with NC and SMYD3 siRNAs. D–G Quantitative statistical results of SMYD3 knockdown in vitro experiments. H SMYD3 mRNA levels in OSCC cells transfected with vector and SMYD3 plasmid. I–K Quantitative statistical results of SMYD3 overexpression in vitro experiments. L The protein expressions of SMYD3 and H3K4me3 were detected after transfection of CAL-27 cell line with SMYD3 plasmids. M, N SMYD3 mRNA and protein levels in CAL-27 transfected with shNC and shSMYD3. *P < 0.05, **P ≤ 0.01, and ***P ≤ 0.001. Fig. S6. BCI-121 suppresses OSCC cells stemness maintenance and proliferatio [file 13148_2023_1506_MOESM1_ESM.zip › SFig 1.tif]

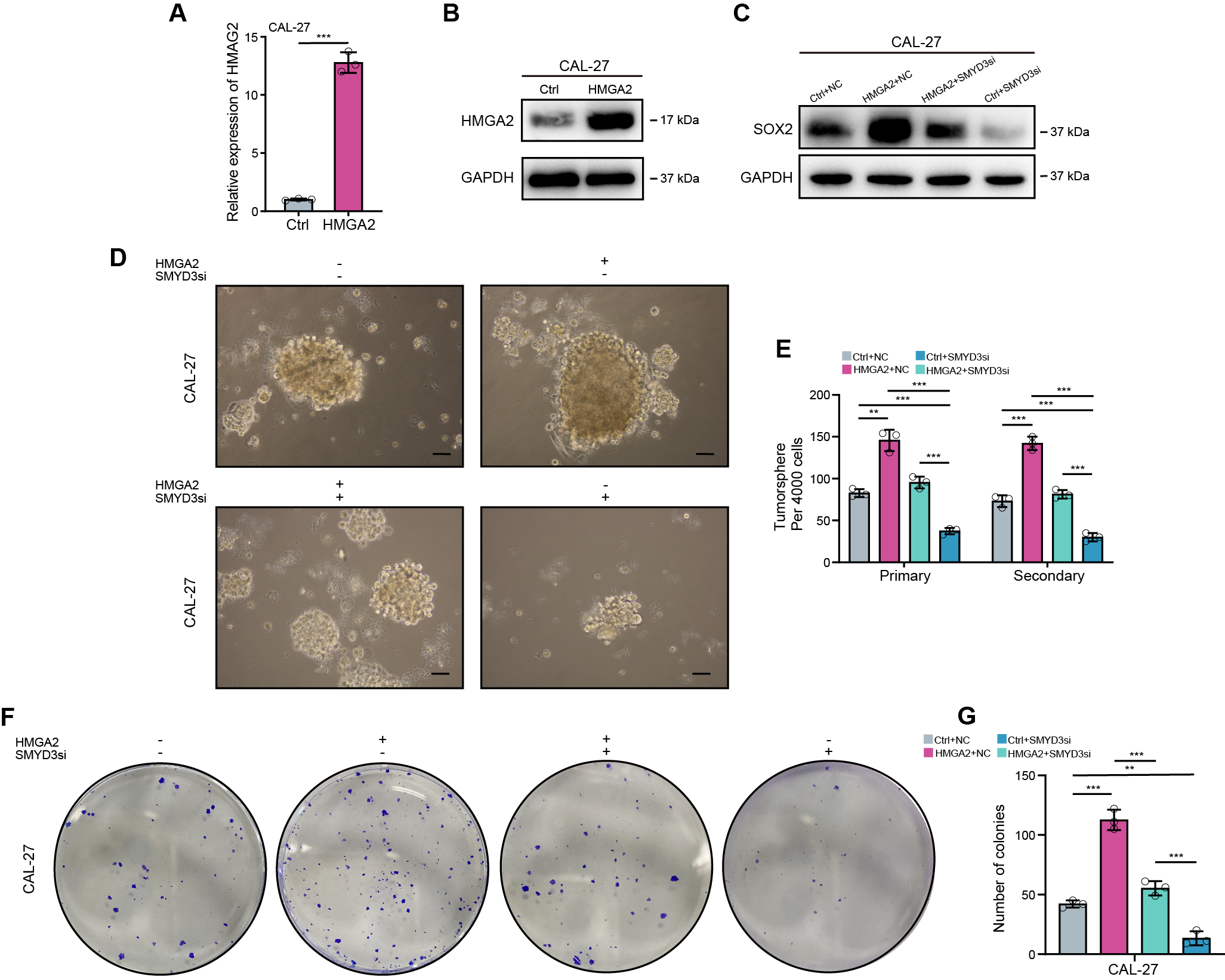

Supplement: Supplementary file 1 — Additional file 1: Fig. S1. Identification of SMYD3 for diagnosis of OSCC. A–E ROC curve analyses of SMYD3 in TCGA, meta-GEO, TCGA, GSE37991, and GSE30784 datasets. AUC values are shown. Fig. S2. The DNA methylation and genomic mutation profile in the TCGA-OSCC dataset. A The correlation of SMYD3 expression and DNA methylation level in TCGA-OSCC cohort. B Value differences of DNA methylation probes in normaland tumortissues from TCGA-OSCC cohort. C The lollipop plot illustrates the differential distribution of somatic mutation in the TCGA-OSCC dataset for SMYD3. D, E ROC curve analyses of SMYD3 in qRT-PCR and IHC staining of collected samples, respectively. Ns, not significant, *P < 0.05, **P ≤ 0.01, and ***P ≤ 0.001. Fig. S3. High expression of SMYD3 indicates increased H3K4me3 modification and HMGA2 expression. A–F IHC images of high and low protein expression of SMYD3, H3K4me3 and HMGA2. Scale bars: 100 μm. Fig. S4. Biological function and pathway enrichment analysis. A The results of GO analysis of RNA-seq on two groups of CAL-27 transfected with NC and SMYD3 siRNA. B The results of KEGG analysis of RNA-seq on two groups of CAL-27 transfected with NC and SMYD3 siRNA. Fig. S5. SMYD3 facilitates OSCC cell stemness maintenance and proliferation in vitro and tumorigenesis in vivo. A, B SMYD3 mRNA and protein levels in CAL-27 and UM-SCC-1 cell lines. C SMYD3 mRNA levels in OSCC cells transfected with NC and SMYD3 siRNAs. D–G Quantitative statistical results of SMYD3 knockdown in vitro experiments. H SMYD3 mRNA levels in OSCC cells transfected with vector and SMYD3 plasmid. I–K Quantitative statistical results of SMYD3 overexpression in vitro experiments. L The protein expressions of SMYD3 and H3K4me3 were detected after transfection of CAL-27 cell line with SMYD3 plasmids. M, N SMYD3 mRNA and protein levels in CAL-27 transfected with shNC and shSMYD3. *P < 0.05, **P ≤ 0.01, and ***P ≤ 0.001. Fig. S6. BCI-121 suppresses OSCC cells stemness maintenance and proliferatio [file 13148_2023_1506_MOESM1_ESM.zip › SFig 10.tif]

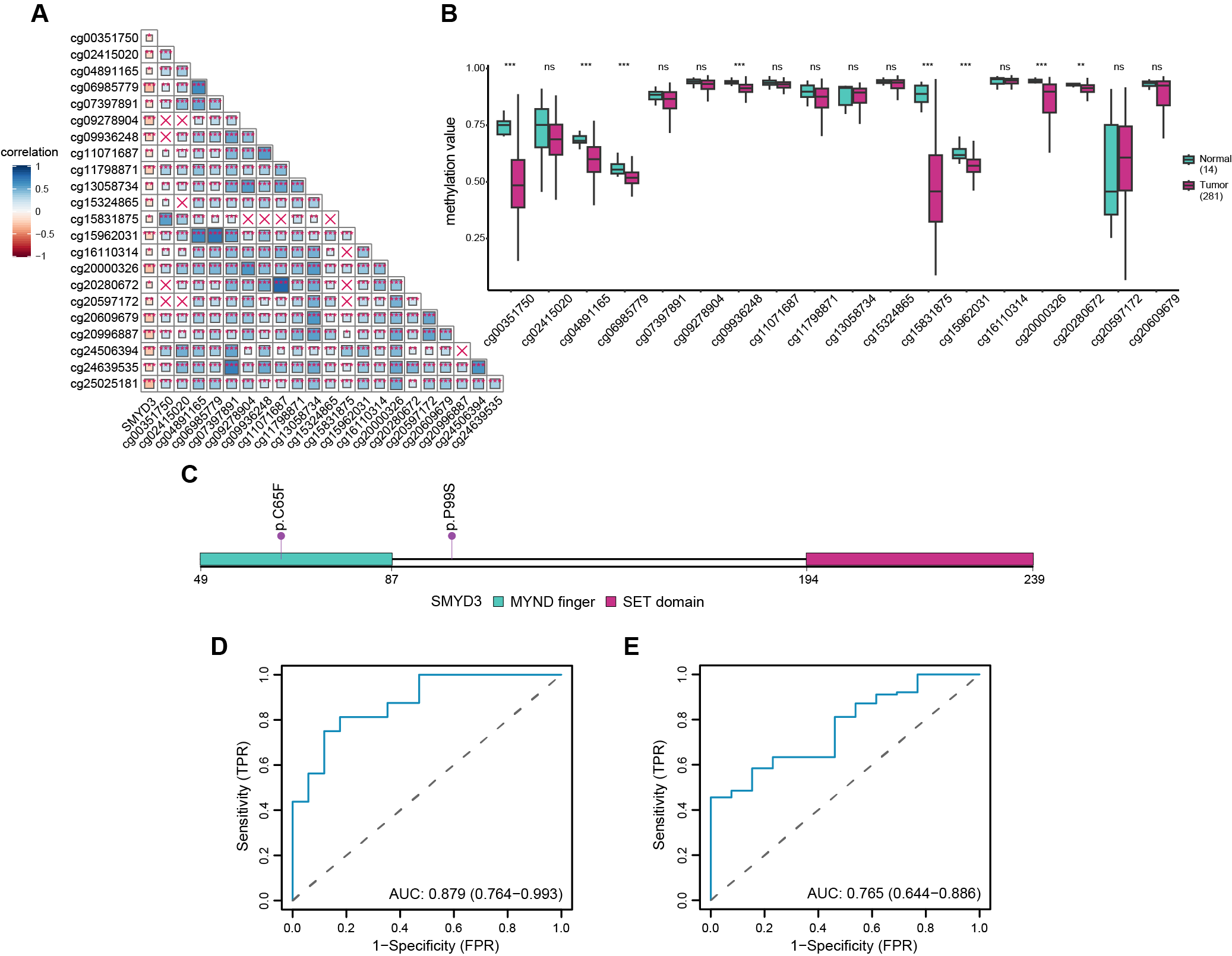

Supplement: Supplementary file 1 — Additional file 1: Fig. S1. Identification of SMYD3 for diagnosis of OSCC. A–E ROC curve analyses of SMYD3 in TCGA, meta-GEO, TCGA, GSE37991, and GSE30784 datasets. AUC values are shown. Fig. S2. The DNA methylation and genomic mutation profile in the TCGA-OSCC dataset. A The correlation of SMYD3 expression and DNA methylation level in TCGA-OSCC cohort. B Value differences of DNA methylation probes in normaland tumortissues from TCGA-OSCC cohort. C The lollipop plot illustrates the differential distribution of somatic mutation in the TCGA-OSCC dataset for SMYD3. D, E ROC curve analyses of SMYD3 in qRT-PCR and IHC staining of collected samples, respectively. Ns, not significant, *P < 0.05, **P ≤ 0.01, and ***P ≤ 0.001. Fig. S3. High expression of SMYD3 indicates increased H3K4me3 modification and HMGA2 expression. A–F IHC images of high and low protein expression of SMYD3, H3K4me3 and HMGA2. Scale bars: 100 μm. Fig. S4. Biological function and pathway enrichment analysis. A The results of GO analysis of RNA-seq on two groups of CAL-27 transfected with NC and SMYD3 siRNA. B The results of KEGG analysis of RNA-seq on two groups of CAL-27 transfected with NC and SMYD3 siRNA. Fig. S5. SMYD3 facilitates OSCC cell stemness maintenance and proliferation in vitro and tumorigenesis in vivo. A, B SMYD3 mRNA and protein levels in CAL-27 and UM-SCC-1 cell lines. C SMYD3 mRNA levels in OSCC cells transfected with NC and SMYD3 siRNAs. D–G Quantitative statistical results of SMYD3 knockdown in vitro experiments. H SMYD3 mRNA levels in OSCC cells transfected with vector and SMYD3 plasmid. I–K Quantitative statistical results of SMYD3 overexpression in vitro experiments. L The protein expressions of SMYD3 and H3K4me3 were detected after transfection of CAL-27 cell line with SMYD3 plasmids. M, N SMYD3 mRNA and protein levels in CAL-27 transfected with shNC and shSMYD3. *P < 0.05, **P ≤ 0.01, and ***P ≤ 0.001. Fig. S6. BCI-121 suppresses OSCC cells stemness maintenance and proliferatio [file 13148_2023_1506_MOESM1_ESM.zip › SFig 2.tif]

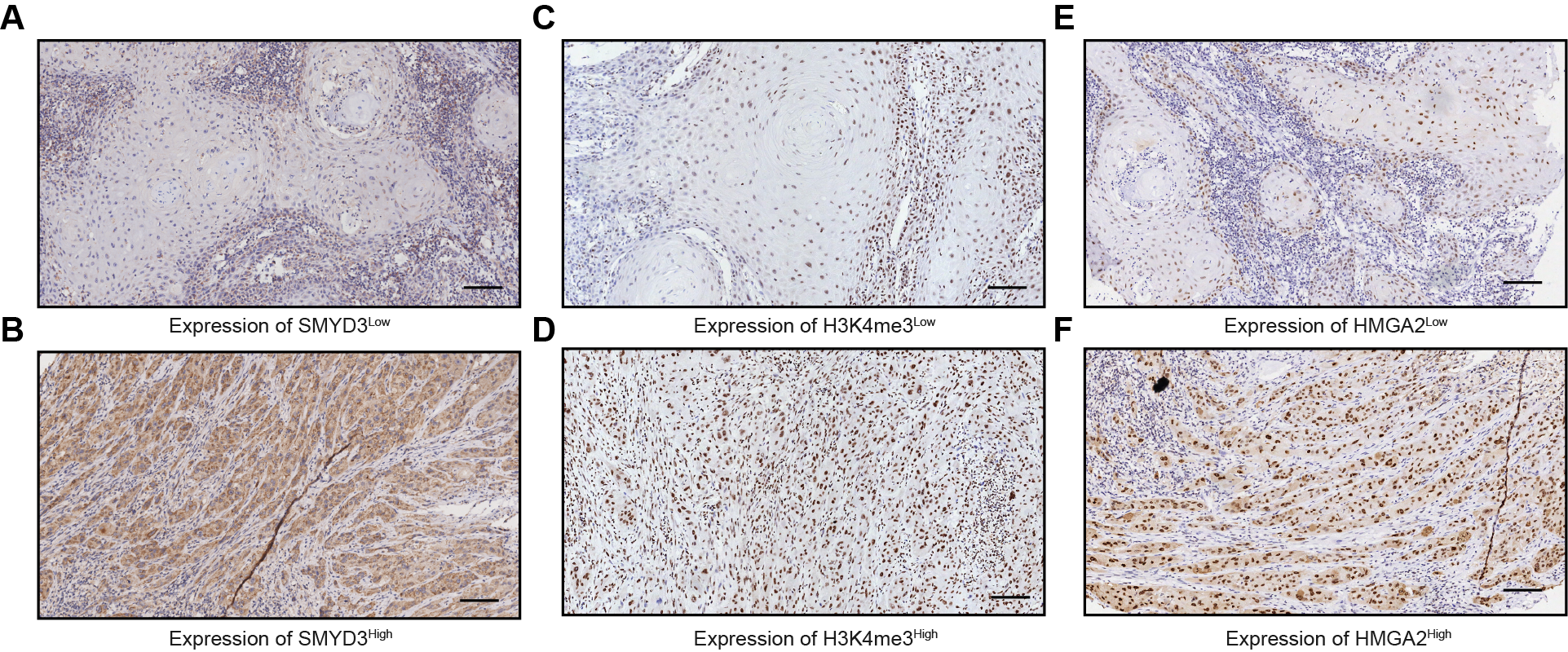

Supplement: Supplementary file 1 — Additional file 1: Fig. S1. Identification of SMYD3 for diagnosis of OSCC. A–E ROC curve analyses of SMYD3 in TCGA, meta-GEO, TCGA, GSE37991, and GSE30784 datasets. AUC values are shown. Fig. S2. The DNA methylation and genomic mutation profile in the TCGA-OSCC dataset. A The correlation of SMYD3 expression and DNA methylation level in TCGA-OSCC cohort. B Value differences of DNA methylation probes in normaland tumortissues from TCGA-OSCC cohort. C The lollipop plot illustrates the differential distribution of somatic mutation in the TCGA-OSCC dataset for SMYD3. D, E ROC curve analyses of SMYD3 in qRT-PCR and IHC staining of collected samples, respectively. Ns, not significant, *P < 0.05, **P ≤ 0.01, and ***P ≤ 0.001. Fig. S3. High expression of SMYD3 indicates increased H3K4me3 modification and HMGA2 expression. A–F IHC images of high and low protein expression of SMYD3, H3K4me3 and HMGA2. Scale bars: 100 μm. Fig. S4. Biological function and pathway enrichment analysis. A The results of GO analysis of RNA-seq on two groups of CAL-27 transfected with NC and SMYD3 siRNA. B The results of KEGG analysis of RNA-seq on two groups of CAL-27 transfected with NC and SMYD3 siRNA. Fig. S5. SMYD3 facilitates OSCC cell stemness maintenance and proliferation in vitro and tumorigenesis in vivo. A, B SMYD3 mRNA and protein levels in CAL-27 and UM-SCC-1 cell lines. C SMYD3 mRNA levels in OSCC cells transfected with NC and SMYD3 siRNAs. D–G Quantitative statistical results of SMYD3 knockdown in vitro experiments. H SMYD3 mRNA levels in OSCC cells transfected with vector and SMYD3 plasmid. I–K Quantitative statistical results of SMYD3 overexpression in vitro experiments. L The protein expressions of SMYD3 and H3K4me3 were detected after transfection of CAL-27 cell line with SMYD3 plasmids. M, N SMYD3 mRNA and protein levels in CAL-27 transfected with shNC and shSMYD3. *P < 0.05, **P ≤ 0.01, and ***P ≤ 0.001. Fig. S6. BCI-121 suppresses OSCC cells stemness maintenance and proliferatio [file 13148_2023_1506_MOESM1_ESM.zip › SFig 3.tif]

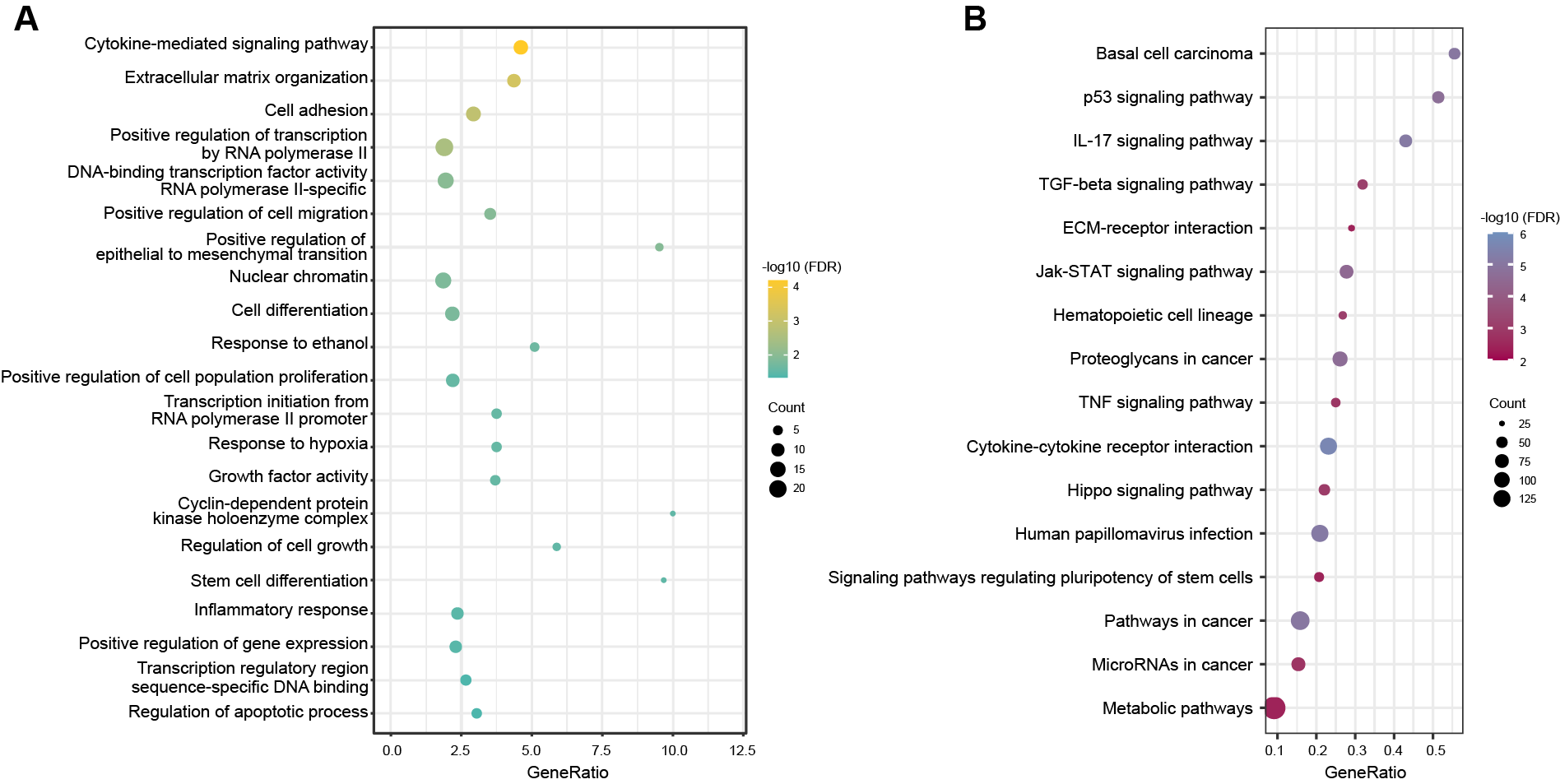

Supplement: Supplementary file 1 — Additional file 1: Fig. S1. Identification of SMYD3 for diagnosis of OSCC. A–E ROC curve analyses of SMYD3 in TCGA, meta-GEO, TCGA, GSE37991, and GSE30784 datasets. AUC values are shown. Fig. S2. The DNA methylation and genomic mutation profile in the TCGA-OSCC dataset. A The correlation of SMYD3 expression and DNA methylation level in TCGA-OSCC cohort. B Value differences of DNA methylation probes in normaland tumortissues from TCGA-OSCC cohort. C The lollipop plot illustrates the differential distribution of somatic mutation in the TCGA-OSCC dataset for SMYD3. D, E ROC curve analyses of SMYD3 in qRT-PCR and IHC staining of collected samples, respectively. Ns, not significant, *P < 0.05, **P ≤ 0.01, and ***P ≤ 0.001. Fig. S3. High expression of SMYD3 indicates increased H3K4me3 modification and HMGA2 expression. A–F IHC images of high and low protein expression of SMYD3, H3K4me3 and HMGA2. Scale bars: 100 μm. Fig. S4. Biological function and pathway enrichment analysis. A The results of GO analysis of RNA-seq on two groups of CAL-27 transfected with NC and SMYD3 siRNA. B The results of KEGG analysis of RNA-seq on two groups of CAL-27 transfected with NC and SMYD3 siRNA. Fig. S5. SMYD3 facilitates OSCC cell stemness maintenance and proliferation in vitro and tumorigenesis in vivo. A, B SMYD3 mRNA and protein levels in CAL-27 and UM-SCC-1 cell lines. C SMYD3 mRNA levels in OSCC cells transfected with NC and SMYD3 siRNAs. D–G Quantitative statistical results of SMYD3 knockdown in vitro experiments. H SMYD3 mRNA levels in OSCC cells transfected with vector and SMYD3 plasmid. I–K Quantitative statistical results of SMYD3 overexpression in vitro experiments. L The protein expressions of SMYD3 and H3K4me3 were detected after transfection of CAL-27 cell line with SMYD3 plasmids. M, N SMYD3 mRNA and protein levels in CAL-27 transfected with shNC and shSMYD3. *P < 0.05, **P ≤ 0.01, and ***P ≤ 0.001. Fig. S6. BCI-121 suppresses OSCC cells stemness maintenance and proliferatio [file 13148_2023_1506_MOESM1_ESM.zip › SFig 4.tif]

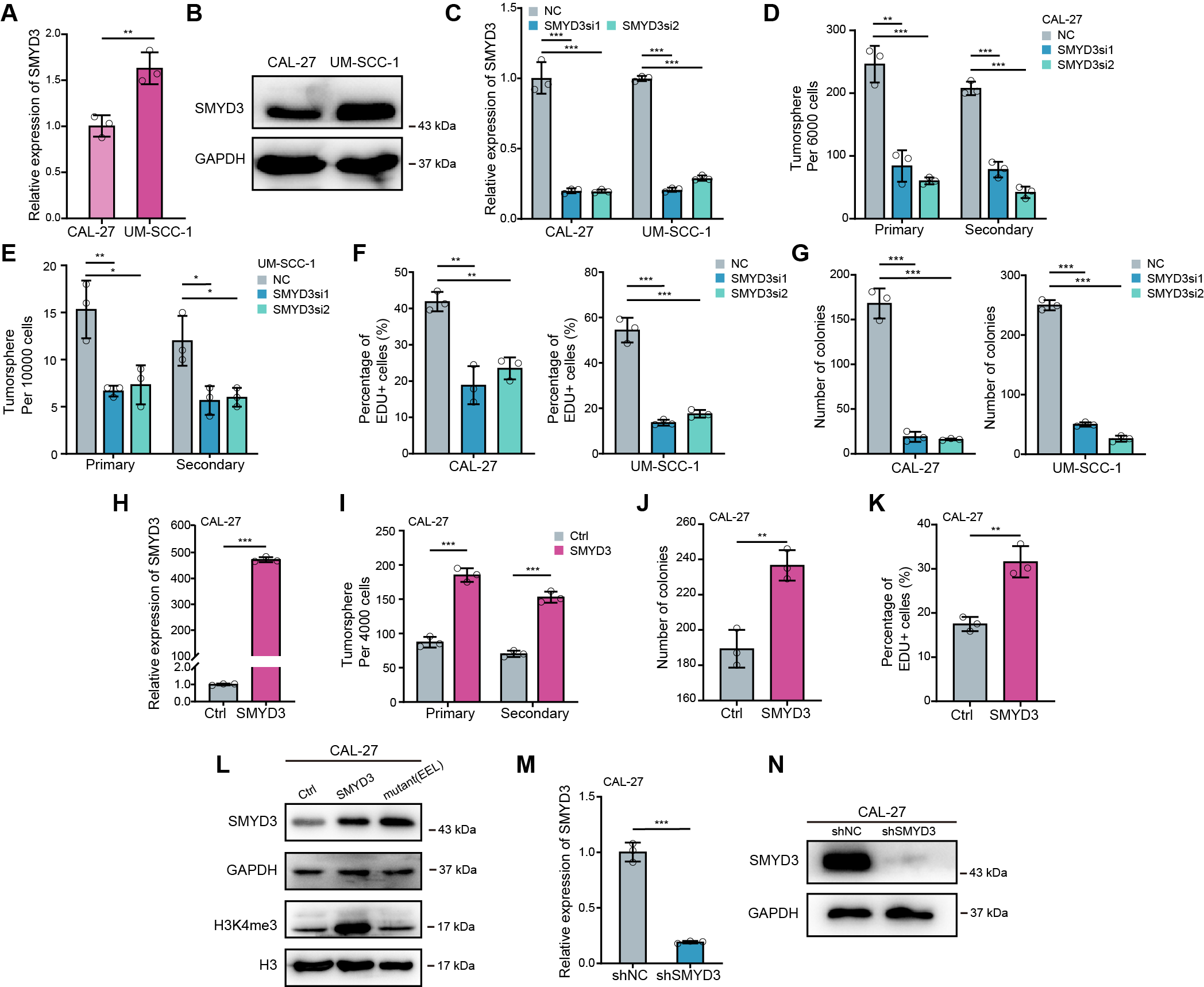

Supplement: Supplementary file 1 — Additional file 1: Fig. S1. Identification of SMYD3 for diagnosis of OSCC. A–E ROC curve analyses of SMYD3 in TCGA, meta-GEO, TCGA, GSE37991, and GSE30784 datasets. AUC values are shown. Fig. S2. The DNA methylation and genomic mutation profile in the TCGA-OSCC dataset. A The correlation of SMYD3 expression and DNA methylation level in TCGA-OSCC cohort. B Value differences of DNA methylation probes in normaland tumortissues from TCGA-OSCC cohort. C The lollipop plot illustrates the differential distribution of somatic mutation in the TCGA-OSCC dataset for SMYD3. D, E ROC curve analyses of SMYD3 in qRT-PCR and IHC staining of collected samples, respectively. Ns, not significant, *P < 0.05, **P ≤ 0.01, and ***P ≤ 0.001. Fig. S3. High expression of SMYD3 indicates increased H3K4me3 modification and HMGA2 expression. A–F IHC images of high and low protein expression of SMYD3, H3K4me3 and HMGA2. Scale bars: 100 μm. Fig. S4. Biological function and pathway enrichment analysis. A The results of GO analysis of RNA-seq on two groups of CAL-27 transfected with NC and SMYD3 siRNA. B The results of KEGG analysis of RNA-seq on two groups of CAL-27 transfected with NC and SMYD3 siRNA. Fig. S5. SMYD3 facilitates OSCC cell stemness maintenance and proliferation in vitro and tumorigenesis in vivo. A, B SMYD3 mRNA and protein levels in CAL-27 and UM-SCC-1 cell lines. C SMYD3 mRNA levels in OSCC cells transfected with NC and SMYD3 siRNAs. D–G Quantitative statistical results of SMYD3 knockdown in vitro experiments. H SMYD3 mRNA levels in OSCC cells transfected with vector and SMYD3 plasmid. I–K Quantitative statistical results of SMYD3 overexpression in vitro experiments. L The protein expressions of SMYD3 and H3K4me3 were detected after transfection of CAL-27 cell line with SMYD3 plasmids. M, N SMYD3 mRNA and protein levels in CAL-27 transfected with shNC and shSMYD3. *P < 0.05, **P ≤ 0.01, and ***P ≤ 0.001. Fig. S6. BCI-121 suppresses OSCC cells stemness maintenance and proliferatio [file 13148_2023_1506_MOESM1_ESM.zip › SFig 5.tif]

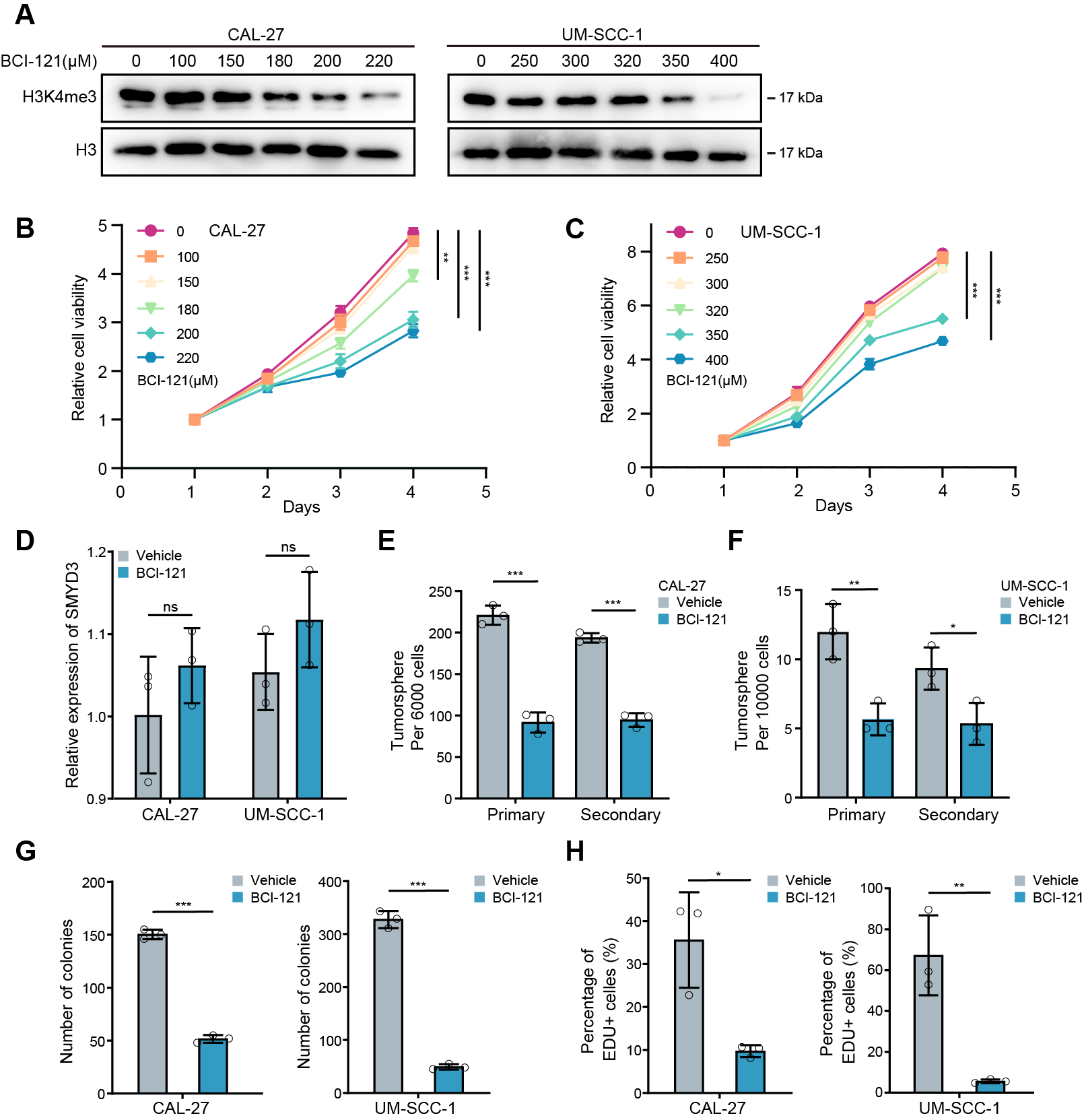

Supplement: Supplementary file 1 — Additional file 1: Fig. S1. Identification of SMYD3 for diagnosis of OSCC. A–E ROC curve analyses of SMYD3 in TCGA, meta-GEO, TCGA, GSE37991, and GSE30784 datasets. AUC values are shown. Fig. S2. The DNA methylation and genomic mutation profile in the TCGA-OSCC dataset. A The correlation of SMYD3 expression and DNA methylation level in TCGA-OSCC cohort. B Value differences of DNA methylation probes in normaland tumortissues from TCGA-OSCC cohort. C The lollipop plot illustrates the differential distribution of somatic mutation in the TCGA-OSCC dataset for SMYD3. D, E ROC curve analyses of SMYD3 in qRT-PCR and IHC staining of collected samples, respectively. Ns, not significant, *P < 0.05, **P ≤ 0.01, and ***P ≤ 0.001. Fig. S3. High expression of SMYD3 indicates increased H3K4me3 modification and HMGA2 expression. A–F IHC images of high and low protein expression of SMYD3, H3K4me3 and HMGA2. Scale bars: 100 μm. Fig. S4. Biological function and pathway enrichment analysis. A The results of GO analysis of RNA-seq on two groups of CAL-27 transfected with NC and SMYD3 siRNA. B The results of KEGG analysis of RNA-seq on two groups of CAL-27 transfected with NC and SMYD3 siRNA. Fig. S5. SMYD3 facilitates OSCC cell stemness maintenance and proliferation in vitro and tumorigenesis in vivo. A, B SMYD3 mRNA and protein levels in CAL-27 and UM-SCC-1 cell lines. C SMYD3 mRNA levels in OSCC cells transfected with NC and SMYD3 siRNAs. D–G Quantitative statistical results of SMYD3 knockdown in vitro experiments. H SMYD3 mRNA levels in OSCC cells transfected with vector and SMYD3 plasmid. I–K Quantitative statistical results of SMYD3 overexpression in vitro experiments. L The protein expressions of SMYD3 and H3K4me3 were detected after transfection of CAL-27 cell line with SMYD3 plasmids. M, N SMYD3 mRNA and protein levels in CAL-27 transfected with shNC and shSMYD3. *P < 0.05, **P ≤ 0.01, and ***P ≤ 0.001. Fig. S6. BCI-121 suppresses OSCC cells stemness maintenance and proliferatio [file 13148_2023_1506_MOESM1_ESM.zip › SFig 6.tif]

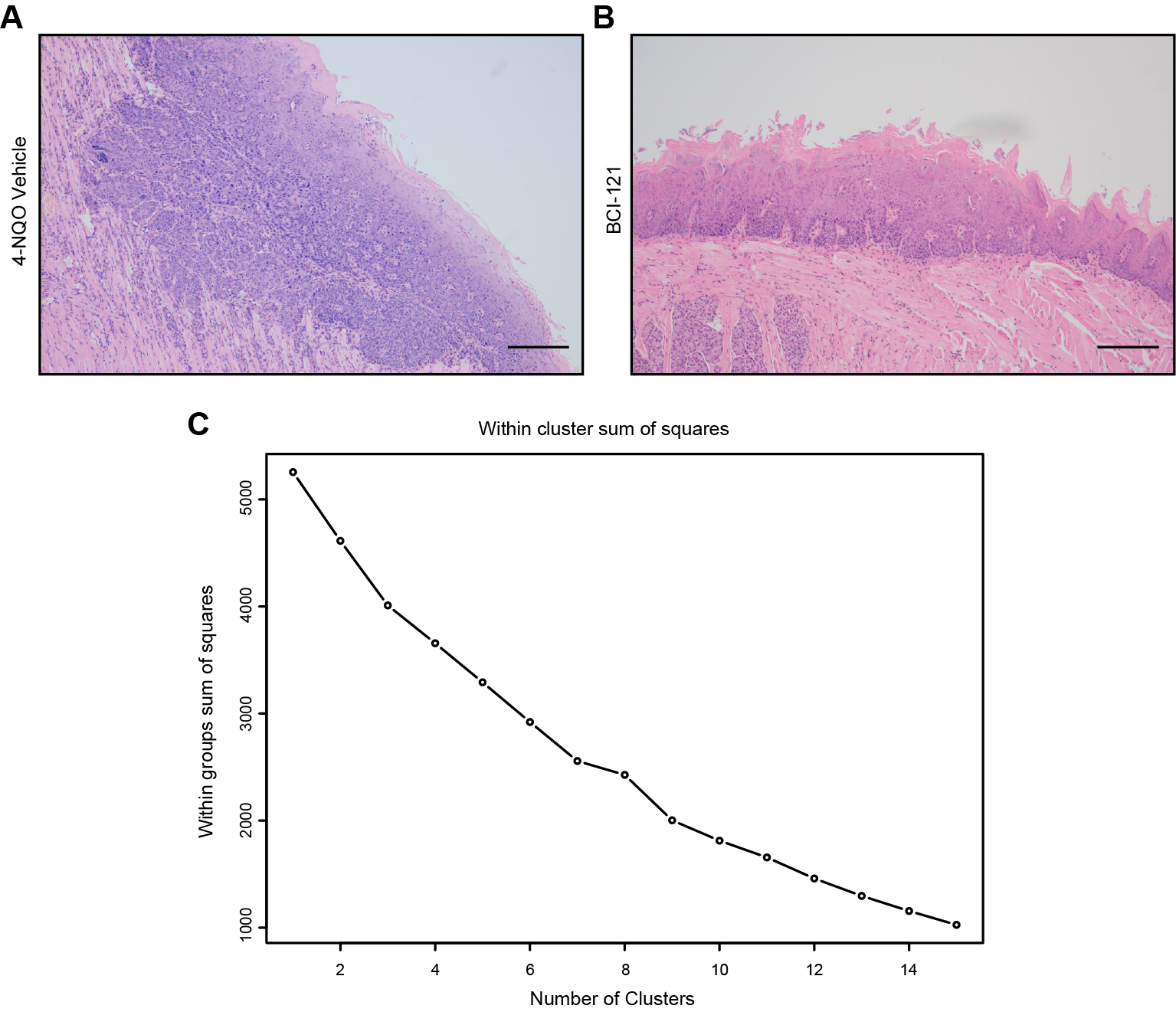

Supplement: Supplementary file 1 — Additional file 1: Fig. S1. Identification of SMYD3 for diagnosis of OSCC. A–E ROC curve analyses of SMYD3 in TCGA, meta-GEO, TCGA, GSE37991, and GSE30784 datasets. AUC values are shown. Fig. S2. The DNA methylation and genomic mutation profile in the TCGA-OSCC dataset. A The correlation of SMYD3 expression and DNA methylation level in TCGA-OSCC cohort. B Value differences of DNA methylation probes in normaland tumortissues from TCGA-OSCC cohort. C The lollipop plot illustrates the differential distribution of somatic mutation in the TCGA-OSCC dataset for SMYD3. D, E ROC curve analyses of SMYD3 in qRT-PCR and IHC staining of collected samples, respectively. Ns, not significant, *P < 0.05, **P ≤ 0.01, and ***P ≤ 0.001. Fig. S3. High expression of SMYD3 indicates increased H3K4me3 modification and HMGA2 expression. A–F IHC images of high and low protein expression of SMYD3, H3K4me3 and HMGA2. Scale bars: 100 μm. Fig. S4. Biological function and pathway enrichment analysis. A The results of GO analysis of RNA-seq on two groups of CAL-27 transfected with NC and SMYD3 siRNA. B The results of KEGG analysis of RNA-seq on two groups of CAL-27 transfected with NC and SMYD3 siRNA. Fig. S5. SMYD3 facilitates OSCC cell stemness maintenance and proliferation in vitro and tumorigenesis in vivo. A, B SMYD3 mRNA and protein levels in CAL-27 and UM-SCC-1 cell lines. C SMYD3 mRNA levels in OSCC cells transfected with NC and SMYD3 siRNAs. D–G Quantitative statistical results of SMYD3 knockdown in vitro experiments. H SMYD3 mRNA levels in OSCC cells transfected with vector and SMYD3 plasmid. I–K Quantitative statistical results of SMYD3 overexpression in vitro experiments. L The protein expressions of SMYD3 and H3K4me3 were detected after transfection of CAL-27 cell line with SMYD3 plasmids. M, N SMYD3 mRNA and protein levels in CAL-27 transfected with shNC and shSMYD3. *P < 0.05, **P ≤ 0.01, and ***P ≤ 0.001. Fig. S6. BCI-121 suppresses OSCC cells stemness maintenance and proliferatio [file 13148_2023_1506_MOESM1_ESM.zip › SFig 7.tif]

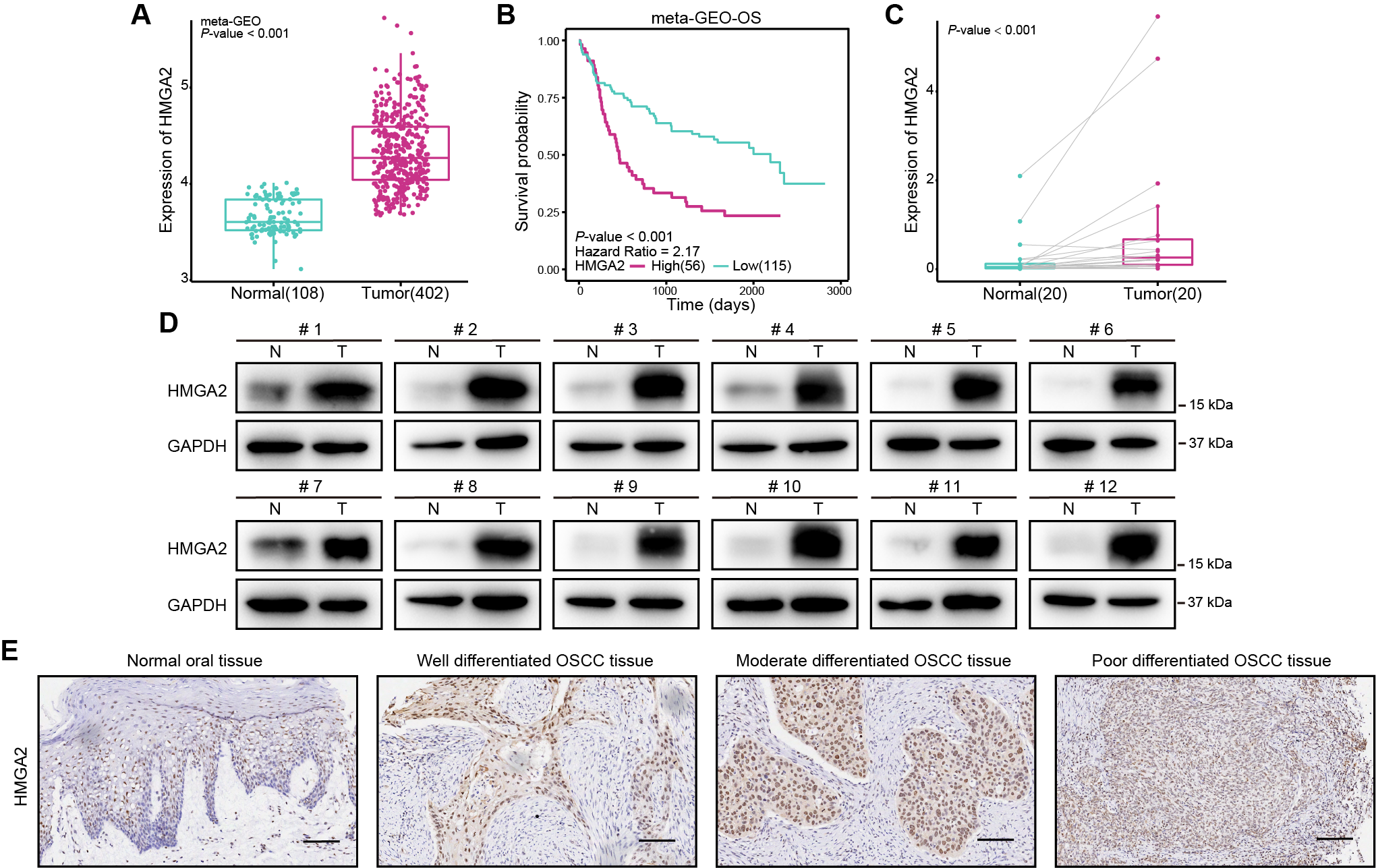

Supplement: Supplementary file 1 — Additional file 1: Fig. S1. Identification of SMYD3 for diagnosis of OSCC. A–E ROC curve analyses of SMYD3 in TCGA, meta-GEO, TCGA, GSE37991, and GSE30784 datasets. AUC values are shown. Fig. S2. The DNA methylation and genomic mutation profile in the TCGA-OSCC dataset. A The correlation of SMYD3 expression and DNA methylation level in TCGA-OSCC cohort. B Value differences of DNA methylation probes in normaland tumortissues from TCGA-OSCC cohort. C The lollipop plot illustrates the differential distribution of somatic mutation in the TCGA-OSCC dataset for SMYD3. D, E ROC curve analyses of SMYD3 in qRT-PCR and IHC staining of collected samples, respectively. Ns, not significant, *P < 0.05, **P ≤ 0.01, and ***P ≤ 0.001. Fig. S3. High expression of SMYD3 indicates increased H3K4me3 modification and HMGA2 expression. A–F IHC images of high and low protein expression of SMYD3, H3K4me3 and HMGA2. Scale bars: 100 μm. Fig. S4. Biological function and pathway enrichment analysis. A The results of GO analysis of RNA-seq on two groups of CAL-27 transfected with NC and SMYD3 siRNA. B The results of KEGG analysis of RNA-seq on two groups of CAL-27 transfected with NC and SMYD3 siRNA. Fig. S5. SMYD3 facilitates OSCC cell stemness maintenance and proliferation in vitro and tumorigenesis in vivo. A, B SMYD3 mRNA and protein levels in CAL-27 and UM-SCC-1 cell lines. C SMYD3 mRNA levels in OSCC cells transfected with NC and SMYD3 siRNAs. D–G Quantitative statistical results of SMYD3 knockdown in vitro experiments. H SMYD3 mRNA levels in OSCC cells transfected with vector and SMYD3 plasmid. I–K Quantitative statistical results of SMYD3 overexpression in vitro experiments. L The protein expressions of SMYD3 and H3K4me3 were detected after transfection of CAL-27 cell line with SMYD3 plasmids. M, N SMYD3 mRNA and protein levels in CAL-27 transfected with shNC and shSMYD3. *P < 0.05, **P ≤ 0.01, and ***P ≤ 0.001. Fig. S6. BCI-121 suppresses OSCC cells stemness maintenance and proliferatio [file 13148_2023_1506_MOESM1_ESM.zip › SFig 8.tif]

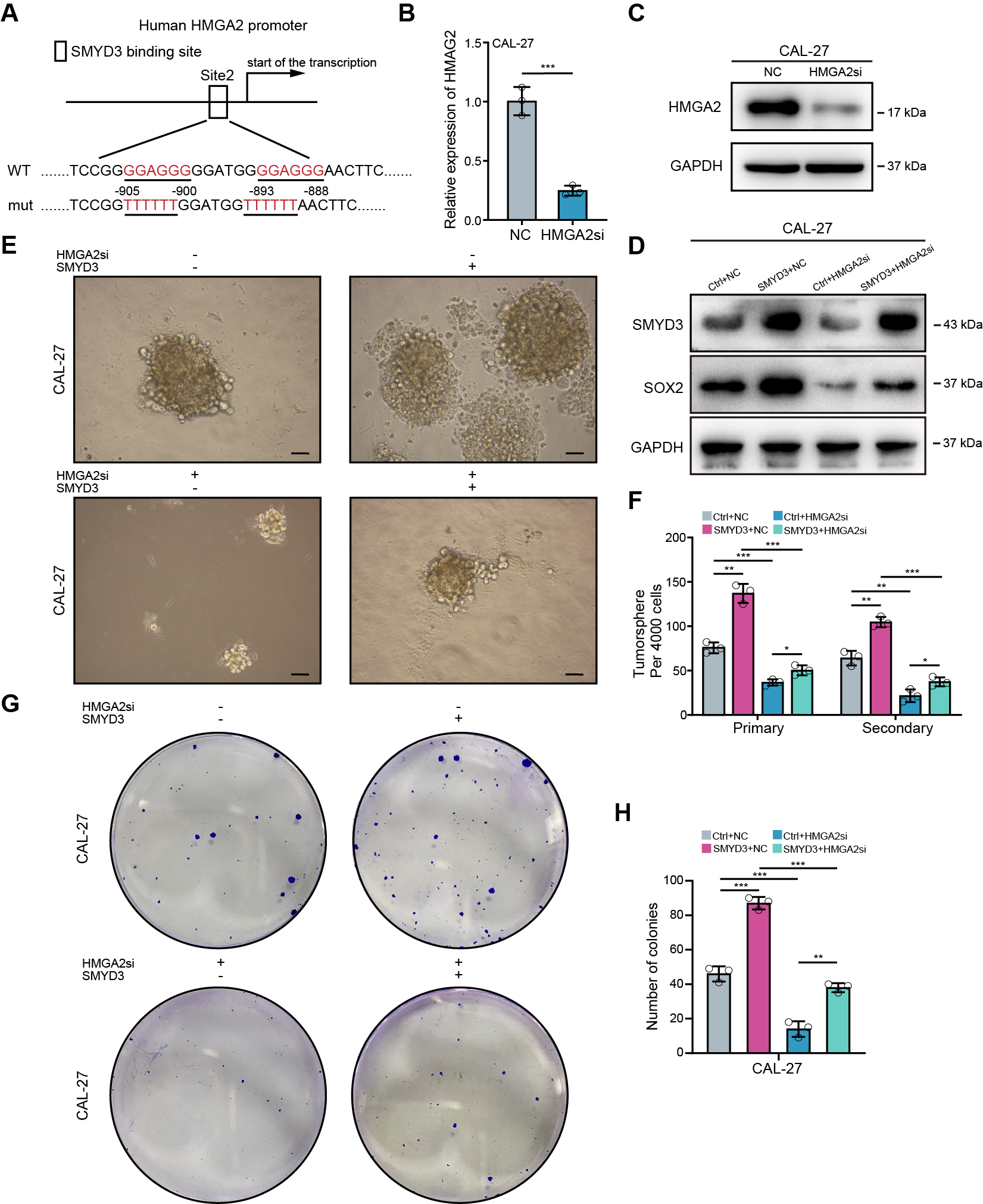

Supplement: Supplementary file 1 — Additional file 1: Fig. S1. Identification of SMYD3 for diagnosis of OSCC. A–E ROC curve analyses of SMYD3 in TCGA, meta-GEO, TCGA, GSE37991, and GSE30784 datasets. AUC values are shown. Fig. S2. The DNA methylation and genomic mutation profile in the TCGA-OSCC dataset. A The correlation of SMYD3 expression and DNA methylation level in TCGA-OSCC cohort. B Value differences of DNA methylation probes in normaland tumortissues from TCGA-OSCC cohort. C The lollipop plot illustrates the differential distribution of somatic mutation in the TCGA-OSCC dataset for SMYD3. D, E ROC curve analyses of SMYD3 in qRT-PCR and IHC staining of collected samples, respectively. Ns, not significant, *P < 0.05, **P ≤ 0.01, and ***P ≤ 0.001. Fig. S3. High expression of SMYD3 indicates increased H3K4me3 modification and HMGA2 expression. A–F IHC images of high and low protein expression of SMYD3, H3K4me3 and HMGA2. Scale bars: 100 μm. Fig. S4. Biological function and pathway enrichment analysis. A The results of GO analysis of RNA-seq on two groups of CAL-27 transfected with NC and SMYD3 siRNA. B The results of KEGG analysis of RNA-seq on two groups of CAL-27 transfected with NC and SMYD3 siRNA. Fig. S5. SMYD3 facilitates OSCC cell stemness maintenance and proliferation in vitro and tumorigenesis in vivo. A, B SMYD3 mRNA and protein levels in CAL-27 and UM-SCC-1 cell lines. C SMYD3 mRNA levels in OSCC cells transfected with NC and SMYD3 siRNAs. D–G Quantitative statistical results of SMYD3 knockdown in vitro experiments. H SMYD3 mRNA levels in OSCC cells transfected with vector and SMYD3 plasmid. I–K Quantitative statistical results of SMYD3 overexpression in vitro experiments. L The protein expressions of SMYD3 and H3K4me3 were detected after transfection of CAL-27 cell line with SMYD3 plasmids. M, N SMYD3 mRNA and protein levels in CAL-27 transfected with shNC and shSMYD3. *P < 0.05, **P ≤ 0.01, and ***P ≤ 0.001. Fig. S6. BCI-121 suppresses OSCC cells stemness maintenance and proliferatio [file 13148_2023_1506_MOESM1_ESM.zip › SFig 9.tif]
